# Rice farmers’ preferences for seed quality, packaging, and source: A study from northern Bangladesh

**DOI:** 10.1371/journal.pone.0306059

**Published:** 2024-06-21

**Authors:** Md. Abdur Rouf Sarkar, Muhammad Ashraful Habib, Mou Rani Sarker, Md. Mahbubur Rahman, Sultanul Alam, Md. Nazmul Islam Manik, Swati Nayak, Humnath Bhandari

**Affiliations:** 1 School of Economics, Zhongnan University of Economics and Law, Wuhan, China; 2 Agricultural Economics Division, Bangladesh Rice Research Institute, Gazipur, Bangladesh; 3 Rice Breeding Innovation Platform, International Rice Research Institute, Los Banos, Laguna, Philippines; 4 Sustainable Impact Platform, International Rice Research Institute, Dhaka, Bangladesh; 5 Seed Marketing Division, Bangladesh Agricultural Development Corporation, Dhaka, Bangladesh; 6 Jute Seed Division, Bangladesh Agricultural Development Corporation, Dhaka, Bangladesh; 7 Research Cell, Bangladesh Agricultural Development Corporation, Dhaka, Bangladesh; 8 Impact, Policy, and Foresight Department, International Rice Research Institute, Dhaka, Bangladesh; Wroclaw University of Environmental and Life Sciences: Uniwersytet Przyrodniczy we Wroclawiu, POLAND

## Abstract

The use of quality seeds is crucial to improve rice yield, food security, and farmers’ livelihoods. The large informal seed system, limited access to quality seeds, and low seed replacement rate challenge increasing rice yield. Despite robust government initiatives to support the seed system, progress has been slow. Besides, the need for farmers’ behavioural change, enhanced coordination, and communication at the local level has not received adequate attention. We investigate rice farmers’ preferences for quality seed, packet sizes, types, and sources, and assess the impact of utilizing good quality seed. We collected quantitative data from 1196 rice farmers in northern Bangladesh in 2019. To identify major factors influencing farmers’ preferences regarding quality seed, packet sizes, packet types, and seed sources, we employed ordered logit and multinomial logit models. Additionally, we used the propensity score matching procedure to assess the impact of good quality and formal seed sources on the rice yield. The findings revealed that farmers strongly prefer using seeds from formal sources despite limited accessibility. Of the total farmers, 34% use public source seeds, 33% use private source seeds, and the rest rely on their own saved seeds. The use of good quality seeds increased rice yields from the base yield by 0.07–0.28 t/ha. We found about a 48% gap in accessing good-quality seeds, indicating significant potential for scaling up the seed systems. Farmers using formal seed sources yielded 0.03–0.15 t/ha more than informal seed users. Farmers strongly prefer 5 kg packets due to their cost-effectiveness, easy storage, and handling convenience. Additionally, farmers prefer *polycoated* jute sacks for their versatility, multi-purpose applications, and resistance to pests. The econometric model results showed that farmers’ preferences were significantly influenced by gender, farm type, crop yield, seed price, market distance, various stakeholders’ advice, and seed supply systems’ constraints. The government should implement policies and programs to strengthen a well-connected seed network in rural areas, promoting inclusivity, and enhancing rice productivity. Besides, farmers’ needs and preferences should be considered in designing and implementing seed-related initiatives to foster sustainable agricultural development.

## 1. Introduction

Seeds form the foundation of food security. Throughout history, the adoption of key agricultural technologies such as seeds, fertilizers, and irrigation, collectively known as ‘Green Revolution Technologies’, has significantly contributed to the transformation of Bangladesh from a food-deficient to a rice sufficient country [1, 2]. Rice cultivation covers 11.7 million hectares, representing 77% of the total cropped area, and yields an annual production of 38.2 million tons [3]. Nonetheless, Bangladesh faces a substantial quality seed gap, potentially decreasing overall productivity. Inadequate availability, limited access, and underutilization of quality seeds pose significant risks to rice yields [4]. For instance, rice cultivation requires approximately 355,308 tonnes of quality seeds, yet the current supply stands at only 232,588 tonnes [5, 6], indicating a 35% gap in demand and supply. Moreover, rapid population growth, rural migration, climate change, and freshwater scarcity collectively present challenges to sustaining rice security [7, 8].

In Bangladesh, the yield gap for most crops exceeds that of other countries due largely to an inefficient seed supply chain heavily reliant on imports to meet quality seed demand [9, 10]. Challenges such as insufficient market linkages, limited seed production capacity, and inadequate quality control mechanisms plague the efficient seed supply chain [11–13]. In addition, delayed policy implementation has hindered the availability of quality seeds, impeded seed trade, and stifled sector growth [2, 9, 14]. Good quality seeds have the potential to increase crop yields by 5–20% [10, 15], yet less than half of the farmers used certified rice seeds [6, 16], with many relying on saved seeds or local market purchases. Formal seed systems face challenges such as inadequate breeding practices, technology gaps, suboptimal production processes, and delivery deficiencies [17]. Counterfeit seeds prevalent in local markets, along with mishandling and storage inadequacies, lead to low germination rates and subpar yields [18–20]. Consequently, the presence of poor-quality seeds and fraudulent activities diminishes farmers’ inclination to invest in formal seeds and complementary inputs, thereby hindering efforts to enhance crop yields and improve food security [20].

Farmers’ seed preference also plays a crucial role in disseminating and adopting agricultural technologies. For example, in Bangladesh, despite the development of 134 modern rice varieties, the adoption rate of these varieties at the farmer-field level remains limited [2, 4, 21]. This limited adoption can be attributed to the absence of a market-driven seed system that fails to consider farmers’ seed preferences. The existing literature highlights various factors influencing farmers’ varietal preferences, such as yield potential, selling price, demographic characteristics, seed quality, production costs, consumption preferences, accessibility to technology, institutional programs, market factors, and environmental conditions [1, 2, 11, 13, 22–25]. Like varietal preferences, rice farmers’ seed preferences exhibit heterogeneity and context-specificity, which remain largely unexplored but are equally crucial for inclusive seed system development in Bangladesh.

To fill this knowledge gap, this study aims to understand farmers’ preferences for rice seed in northern Bangladesh, specifically regarding quality seed, packet type, packet size, and seed source. This study also identifies the factors influencing rice farmers’ seed preferences and evaluate the impact of good quality and formal seed sources on the rice yield. Our paper departs from earlier literature in the following ways. First, to the best of our knowledge, this is the first study to provide econometric assessment on the identification of the key drivers of rice farmers’ preferences on seed quality, packaging, and source based on farm-level data combined. Second, our study enriches the limited literature on farmers’ investment portfolios in relation to the marketing strategy for rice seed packaging and labeling. Third, the propensity score matching method is employed to estimate the comprehensive impact of good quality seed usage and access to formal seed sources on the rice yield. Overall, through this research, we hope to fill the knowledge gap on rice farmers’ seed preferences and provide evidence-based recommendations to improve seed availability and accessibility in northern Bangladesh. This study can help inform the development of effective strategies to increase farmers’ access to quality seeds, ultimately contributing to improving rice yields, food security, and income generation for farmers in the region.

This article consists of seven sections. Following this introduction, the second section summarizes the literature gaps. The third section explains the methodology and the fourth section presents the study findings. The fifth section discusses the research findings while the sixth section addresses policy considerations, study limitations, and future prospects. The conclusion is presented in the final section of the article.

## 2. Visualization of existing content areas and research gap

**Fig 1:**
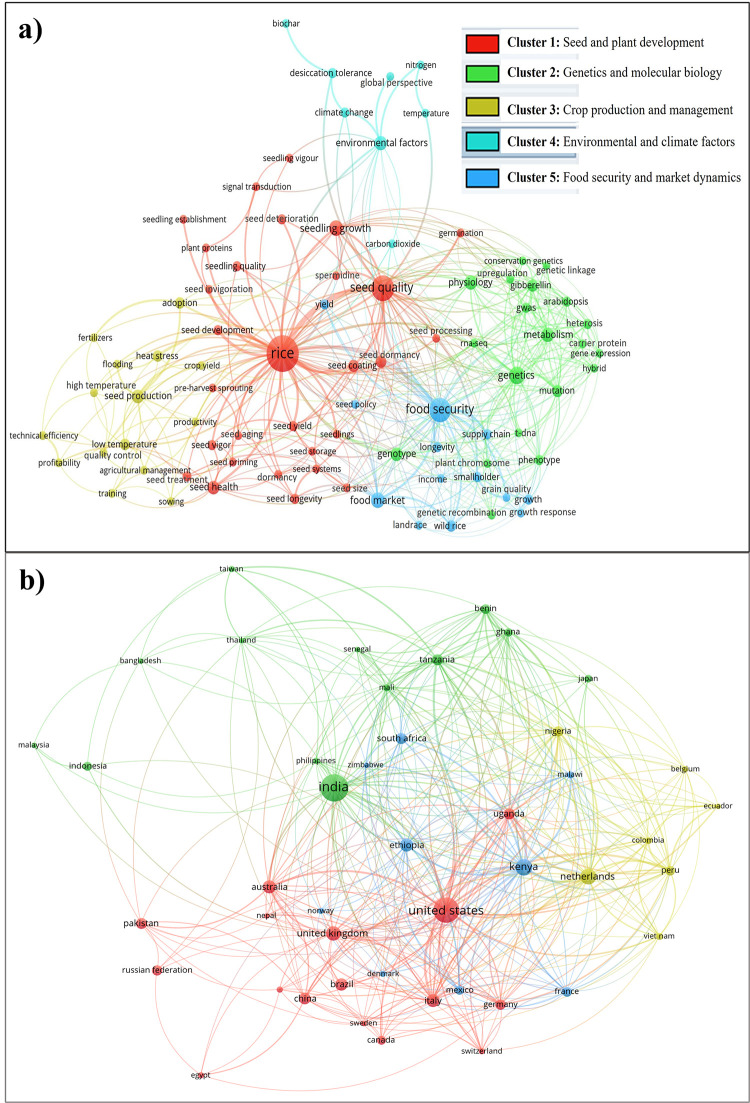
Visualization of the research gap identification process using (a) co-occurring keywords in seed systems studies, and (b) cross-country collaboration.

To identify the existing research gaps in the field of seed system and quality seed of rice, a thorough search was conducted in the Scopus database using targeted keywords including ‘seed system*’, ‘quality seed’, ‘seed source*’, ‘seed packet’, ‘seed quality’, ‘seed production’, ‘seed supply’, ‘seed policy’, and ‘Rice’. The search yielded five prominent clusters, namely seed and plant development, genetics and molecular biology, crop production and management, environmental and climate factors, and food security and market dynamics (Fig 1A). In seed and plant development, studies focus on understanding and improving seed aging, dormancy, health, priming, and coating to enhance seed quality and vigor, ensuring optimal growth and yield. Genetics and molecular biology research delves into genetic components and processes, utilizing advanced techniques like gene expression analysis and genome-wide association studies to improve breeding programs and develop high-yielding hybrids while preserving genetic diversity. Crop production and management research aims to optimize agricultural practices, including seed production, fertilizer use, and stress management, to enhance productivity and profitability. Environmental and climate factors research address the impacts of climate change, soil fertility, and extreme weather conditions on rice seed production, promoting sustainable farming practices. Finally, food security and market dynamics research focuses on optimizing the seed supply chain, ensuring food security, improving farmer incomes, and preserving genetic diversity through robust seed policies, meeting the demands of growing populations and supporting smallholders.

The majority of the existing research was concentrated in Europe, North America, some African countries, and India of the Asian countries, as depicted in Fig 1B. However, the literature about Bangladesh, where rice seed system has primarily focused on limited aspects, including seed germination [26–28], seed quality [29], seed health [28, 30, 31], genetic resource management [32], seed processing [33, 34], seed supply systems [35], seed entrepreneurship [16], and seed policy [9].

We also summarized the extensive body of research on rice seed systems in Table 1, organized into 13 distinct themes. The first theme addresses seed germination and quality, with studies exploring innovative techniques for seed storage and drying, including the use of packaging materials to maintain high germination rates and seed quality [33, 34, 36, 37]. The next theme studies focus on stress tolerance in seedling growth and survival [26]. Bhuiyan et al. [30] studied on seed systems investigates the seed-borne diseases that affect crop yields, as well as cost-effective disease management strategies. However, most studies concentrate on seed supply chains. These studies examine the farmers’ seed channel, issues related to genetic purity, and the roles of both formal and informal channels in meeting national food demand [24, 38–41]. The subsequent literature review delves into diverse facets of farmers’ interactions with seed access, adoption [42], and perceptions within distinct agricultural settings [43, 44]. Next theme center on the factors influencing farmers’ decisions regarding seed quality, availability, and access. Evidence focuses on the economic and social dynamics influencing seed adoption [45, 46]. In the subsequent theme, studies concentrated on farmers’ behavioral patterns within seed system dynamics [47], with a continued focus on farmers’ willingness to pay (WTP) for seeds [48].

**Table 1:** Summary of selected global studies on rice seed systems.

SN	Research theme	Location	Research objective	Key findings	Reference
1	Seed germination and quality	Ethiopia	Different seed packaging materials for quality of rice seed stored	• Airtight bags like PICS and Grain-pro super bags shows higher germination rates, seedling growth, and vigor compared to jute and polypropylene bags.	Assaye et al. [36]
2		Mozambique	Compare the effectiveness of traditional raffia bags and hermetic storage in terms of quantitative losses and seed quality	• Hermetic storage demonstrated a lesser decline in germination potential, within the range of 13.9–17.5%, compared to the significant 38.25% drop observed in traditional storage. • Hermetic storage reduces insect pest infestation while ensuring that germination power and seedling vigor remain above minimum levels.	Guenha et al. [37]
3		Bangladesh	Effects of physiological age on the temperature responses	• Younger seeds show better low temperature tolerance compared to older seeds due to higher vigor. • Seeds that fail to germinate at low temperatures typically begin germination upon transfer to 21°C.	Ali et al. [27]
4		Bangladesh	Effectiveness of two- stage drying technique for quality paddy seeds	• The first option was fluidized bed drying at 50°C, 55°C, and 60°C followed by sun drying at 28–32°C. The second option fluidized bed drying at the same initial temperatures, followed by drying at 35°C, 40°C, and 45°C. • The proposed two-stage drying technique not only reduce the drying time but also enhances the germination capacity of paddy seed samples when dried at optimal air temperatures, compared to the sun-drying method.	Islam et al. [34]
5		Bangladesh	Effect of manual cleaning on seed germination	• Manually cleaned seeds exhibited an average 78% increase in germination.	Mathur et al. [28]
6		Bangladesh	Technical performance and seed quality of the S4S solar grain dryer for paddy seeds	• S4S showed significantly lower average drying compared to open sun drying. • In the Aman season, the S4S demonstrated lower average drying loss compared to sun drying. • The germination rates and vigor index of paddy seeds dried with the S4S were satisfactory, ranging from 82% to 97% and 1638 to 2232 respectively, compared to sun-dried seeds which ranged from 79% to 80% and 1738 to 1973, respectively.	Roy et al. [33]
7	Seed priming and stress tolerance	Bangladesh	The efficacy of various seed priming agents in alleviating cold stress	• Priming proves effective in enhancing seed germination, promoting robust seedling growth, and improving seedling survival rates in winter rice exposed to cold stress. • Potassium chloride (KCl) at 20000 ppm or calcium chloride (CaCl2) at 20000 ppm serving as promising priming agents.	Anwar et al. [26]
8	Seed health and disease management	Bangladesh	Use of botanicals/plant extracts in controlling seedborne pathogens	• Fungicides outperformed plant extracts, but plant extracts offer cost-effective seed-borne infection control for farmers.	Bhuiyan et al. [30]
9		Bangladesh	Role of seed health on crop production	• About 490 seed-borne diseases are identified, affecting the seed health of 76 significant crops, resulting in annual yield losses estimated at Taka 9500 million ($250 million).	Rashid et al. [31]
10	Seed systems and supply chains	Tanzania	Assess rice seed quality collected from formal and informal channels	• Most farmers use their own saved seeds. Around 92% of seeds from informal sources and 80% from formal sources meet minimum national quality standards. • Only one fifth of the seed both systems met the minimum requirement for genetic purity.	Gebeyehu et al. [40]
11		Liberia	Rice seed systems, methods of storing harvested rice grains	• About 95% of farmers used seed from informal system. • Grain for future planting is predominantly stored in kitchen attics (83.8%) followed by plastic containers (7.8%), nylon sacks (3.8%), and jute bags (4.6%), respectively.	Dorley et al. [38]
12		Guinea	Organizational settings of the rice seed sector	• Seed interventions of the country relied on the national extension system, research institutes, NGOs, farmers’ associations, and contract seed producers. • Local seed dealers mostly provide seed to farmers, and governmental organizations pay no attention to their role.	Okry et al. [57]
13		Bangladesh	Causes of limited quality cereal seed supply and suggest policy revisions to improve availability at affordable prices for farmers	• Limited infrastructure, oversight in farmer perceptions, inadequate land data, private sector seed marketing, lack of demand forecasting, high seed prices, delayed payments to contract growers, regulatory constraints of seed certified agency, and absence of effective production plans lead to inadequate quality seed supply. • Policies should enhance quality cereal seed availability through strengthening BADC and SCA, supporting private seed sectors, subsidies, updated statistics, effective production and marketing plans, fair pricing, and seed policy amendments.	Hossain and Ahmed [14]
14		Bangladesh	Seed system development	• Farmers have traditionally depended on their practices to choose, preserve, and trade seeds with favorable genetic traits. • Despite its proven benefits, funding in agricultural R&D, particularly for cultivar improvement, remains insufficient. Moreover, with existing pluralistic formal extension system, the DAE has minimally influenced the dissemination of new cultivar information to farmers.	Naher and Spielman [39]
15		Bangladesh	Rice seeds value chain analysis	BADC produces foundation seed on its farms and provides it to contract growers, who then produce certified seed. • Seed dealers and traders experience higher profit margins. • Women’s involvement in the value chain is limited. • The main challenges were the insufficient capabilities of both the public and private sectors, the lack of modern infrastructure, and a skilled workforce in producing, processing, storing, and marketing high-quality rice seed, along with ineffective policy implementation.	Tulachan et al. [41]
16		Malawi, Kenya, DR Congo, South Sudan, and Zimbabwe	Seed systems of smallholder farmers use	• Smallholder farmers obtained 90.2% of their seed from informal systems, with 50.9% sourced from local markets. • Smallholders invest significantly, with 55% of seeds bought with cash. • Farmers receive 52% of new crop varieties through one-time aid from NGOs or governments. • Historically, investment has mainly targeted the formal public or private sectors over the informal seed system.	McGuire and Sperling [24]
17		South Asia and sub-Saharan Africa	Impact of climate factors on seed production	• Agricultural production could decline by 4–10% under different socioeconomic and climate change scenarios. • Challenges such as shifts in planting seasons, altered precipitation patterns, and increased pest and disease pressures, all of which pose significant risks to seed quality and production. • Changing climates affect seed quality and increase production costs due to factors like crop-weed interactions and loss of pollinator biodiversity. • Intellectual property rights, genetically engineered seeds, new pests, diseases, and declining pollinator and genetic diversity impact seed production quality.	Singh et al. [50]
18	Farmers’ seed access and adoption	Nigeria	Determinants of farmers’ access to certified rice seed	• About 63% of farmers obtain seeds from their own farms. vFarmer’s access to seed significantly increased by factors like extension contact, media, household head’s age, and formal education.	Awotide et al. [60]
19		India	Determinants of farmers’ seed choice	• Farmers who used externally sourced seeds achieved higher yields and greater income than those who used their own seeds. • Farmers’ seed selection was influenced by factors such as economic status, social group, variety awareness, and past experiences with crop loss.	Devi et al. [43]
20		Afghanistan	Assessment of variety and seed need	• Half of households saved their own seed, 32% bought locally, 23% sourced from other farmers, and 5% from aid agencies. • The adoption rate of new varieties remains low. Despite having adequate planting material, the primary issue identified was the poor quality of the seeds used by farmers.	Kugbei et al. [42]
21		Thailand	Farmers’ perception on rice seed production	• Farmers has sufficient knowledge of seed production standards, and nearly 60% followed the right production practices. • The constraints identified are the high cost of inputs and fertilizer, the low rice, and climate variability.	Kummanee et al. [44]
22	Economic and social factors in seed adoption	Global	Households’ preferences and needs for seed systems development	• The demand for seeds and varieties by farming households is shaped by multiple factors. These include demographic changes, technological progress, infrastructure development, economic conditions, environmental factors, social dynamics, and political contexts. • Setting goals for breeding programs required consideration of the inherent heterogeneity among farming households.	Mausch et al. [61]
23		Bangladesh	The significance of grain moisture measurement	• An average annual paddy storage loss of 52 kg or 563 Taka (US$6.78). • Farmers who purchased moisture meters typically had larger farms, were younger, more educated, and possessed off-farm income sources.	Akter et al. [29]
24		Indonesia	Socio-economic factors influencing the adoption of quality seeds in lowland rice farming and fertilizer usage on these seeds	• Education, access to credit, income streams, extension services, and participation in farmer group meetings positively correlate with the adoption of quality seeds and fertilizer usage on these seeds. • Conversely, household size, farm size, and gender exhibit negative associations with fertilizer application on quality seeds.	Effendy et al. [45]
25		India	Farm-level varietal diversity, seed source, and adoption dynamics of rice seed	• About 63% of farmers source seeds from their own farms. • Upland areas exhibit greater varietal diversity then low-lying areas. • Higher landholding sizes and participation in varietal demonstrations correlate with increased diversity levels. Yield, grain quality, cooking quality, diseases, and pest tolerance are affecting variety adoption.	Nayak et al. [46]
26	Behavioral dynamics of seed systems	Nigeria	Farmers’ willingness to pay (WTP) for seeds purchase timing	• Timing and WTP is statistically insignificant for rice seed. Although, rice farmers tend to buy seeds earlier than maize and cowpea farmers, likely due to the relative ease of storing rice.	Takeshima and Nagarajan [48]
27		Vietnam	Determinants of farmers’ adoption and willingness to pay for certified aromatic rice seed (CARS)	• The adoption decision of CARS was motivated by membership in an agricultural organization, relative profitability, a lower seed rate, and seed availability. • The current market price of CARS was higher than willingness-to-pay.	Pham and Napasintuwong [47]
28	Farmer resilience and adaptation	Vietnam, Uganda, Zambia, Niger, and Guatemala	Strengths and weakness of seed producer group	• The groups received support from governments, public institutions, national seed companies, and development organizations. Some sold under contract to institutional buyers, while others sold locally. • To ensure seed quality and effective branding, many groups had subgroups for crop monitoring, seed inspection, and market intelligence. • Groups challenges include timely access to quality early generation seed (EGS) and poor packaging and labeling in local markets.	Dey et al. [49]
29		Uganda	Resilience of farmer seed systems to climate-induced stresses	• Drought ranked top among climate factors affecting crop production, impacting seed systems by reducing yield quantity and quality, leading to decreased seed saving and increased grain prices. • Farmers shifted from farm-saved seed to social networks and local markets during stress period, with local seed businesses emerging as an alternative source.	Kansiime and Mastenbroek [22]
30		Asia, Latin America, Africa and Europe	Opportunities and vulnerabilities within seed systems aids in identifying potential resilience challenges	• Farmers’ seed systems suffer challenges from both sudden shocks (like disasters) and gradual ecological and market change. Commercial formal seed systems are less vulnerable but serve only those farmers who can afford quality seeds. • Formal seed systems face vulnerabilities such as long-term investment needs in breeding, limited government seed production schemes, and inadequate quality control measures.	Louwaars and Manicad [23]
31	Seed quality and farmer efficiency	Afghanistan	Impact of the village-based seed enterprises (VBSEs) to quality seed access	• By fostering trust and cooperation among local farmers, VBSEs enhance the dissemination of quality seeds, improve community resilience. • All VBSEs demonstrated a robust returns-to-asset ratio of 4%, well below the acceptable stress threshold of 6%.	Srinivas et al. [52]
32		Sri Lanka	Impact of seed sources on rice farmers’ technical efficiency (TE)	• Only 18% of farmers used their own saved seeds, while the majority bought seeds from the market. • About 46% grew both hybrid or modern and traditional rice varieties. • The ’seed source’ variable was positively linked to TE, showing that households using only saved seeds were less efficient than those purchasing seeds.	Suresh et al. [51]
33	Training and knowledge dissemination	Nepal	Impact of traders’ meetings (TMs) to promote new rice varieties	• TMs significantly enhance traders’ knowledge and increase the likelihood of selling newly promoted rice varieties. However, TMs did not lead to a significant increase in the overall sales of these promoted rice varieties.	Thapa et al. [53]
34	Gender dynamics in seed systems	India	Gender differences in training and knowledge dissemination in promoting of new rice varieties	• The effectiveness of training sessions in promoting the use of salt-tolerant high-quality seeds, enhancing farmers’ knowledge, and facilitating the adoption of climate-resilient and stress-tolerant rice varieties, along with the utilization of IRRI super bags, was particularly pronounced among female farmers then male farmers.	Dar et al. [54]
35		India	Analyzing seed flow across gender, ethnicity, and age intersections to understand formal and informal supply routes	• Agrobiodiversity emerges as a gendered social-ecological phenomenon. • Informal and formal seed systems exist together and overlap due to the movement of actors within systems. • Women often playing central roles in seed selection, conservation, and management within informal networks.	Schöley and Padmanabha [62]
36		India	Role of social capital of self-help groups for strengthening seed systems	• Women, through collaboration, successfully produced and distributed high-quality rice and wheat seeds to over 30,000 small-scale farmers, actively participating in all stages of the seed value chain and utilizing their social networks to promote the adoption of new varieties.	de Boef et al. [55]
37		Bangladesh	Status of women-led community-based rice seed entrepreneurship model (CBRSEM) and potential benefits	• CBRSEM membership improved communication, social ties, and decision-making skills, while also enhancing agricultural practices like sowing and harvesting. • Access to quality seeds boosted yields and livelihoods, but challenges included high production costs and the absence of a moisture meter.	Nuruzzaman1 et al. [16]
38	Institutional and policy influence	Bangladesh	Assess seed policy reforms and analyze their impact on cereal crop productivity	• The national seed policy has drawn private sector involvement in importing, marketing, and distributing seeds, potentially enhancing crop productivity, yet it encounters sustainability challenges. • Dependence solely on imported seeds for quality may hinder the private sector’s market share expansion. • Except for hybrid rice, policies to attract private sector R&D investments are absent.	Kolady and Awal [9]
39		Nepal	Impact of contract farming (CF) in high yielding varieties of paddy seed production on costs, yield, and profits of smallholder farms	• CF had the potential to increase total profits by NRs. 13,000 and boost yields by approximately 338–358 kg/ha. Additionally, CF reduced total production costs by about NRs. 292 per hectare. • Across all categories of farm size, smaller farms (60.43 ha) experienced a higher degree of benefit.	Mishra et al. [56]
40		Nepal	Role of agricultural extension and support services in developing the seed sector	• There was a notable gap in implementing seed policies and enforcing the legislation. • The seed supply system is mostly informal, lacking proper laboratories, skilled personnel, and alignment with international standards. • Despite enforced seed legislation and a diverse agricultural extension system, the seed replacement rate is very low.	Pokhrel [12]
41		Vietnam	Impacts of seed clubs in ensuring local seed systems	• Nearly 80% of farmer-seed producers bought high-quality seeds from seed centers and research institutes, while some purchased from seed clubs and local traders. • Certified seed prices in the formal system were about 26% (2,000 VND/kg) higher than those from seed club members.	Tin et al. [63]
42	Agrobiodiversity and conservation	India	Role of information seed systems in fostering landrace diversity and on-farm conservation	• About 96% of the seed supply by the informal system. • At higher elevations, landrace cultivation predominates, leading to greater diversification in traditional production. • Environmental adaptations yield valuable germplasm variations for crop improvement purposes.	Pandey et al. [58]
43		India	Nature of transition taking place in rice seed provisioning	• The capacity of private sector seed enterprises is increasing as the seed system is going through a transition from the public to the private sector, which is very slow in the rice sector. • The private sector playing a parastatal role with the public provisioning of rice seeds remains the dominant mechanism.	Pandey et al. [64]
44		Italy	Role of seed sector for the agri-food production	• Favourable environment and high professional skills of companies and farmers played significant role of growing seed crops. • Export and multiplication of seeds are major source of income.	Nardi [59]
45		Bangladesh	Impact of the international rice gene bank’s (IRG) on rice farming	• Access to the diverse genetic resources stored in the IRG has facilitated the development and dissemination of improved rice varieties. • With 52% of improved rice varieties’ genetic composition attributed to IRG accessions, each 1% increase in IRG contribution leads to a 0.99% yield increase, translating to an extra US$8,576,973 in aggregated net benefits for farmers during the wet season.	Villanueva et al. [32]

Research on the theme of climate change and seed production mainly explored how climate-induced stress changes the farmers’ perception and influences the seed channel transition, as well as the impact of climate factors on seed production and quality [22, 49, 50]. In the theme of seed quality and farmers’ efficiency, studies center around the impact of seed enterprises and seed sources on agricultural outcomes [51, 52]. Few focused on the role of social capital by fostering trust and cooperation among local farmers to enhance access to quality seeds, as well as enhance community resilience [51, 52]. Furthermore, Thapa et al. [53] studied the impact of knowledge dissemination in increasing sales and acceptance of promoted cultivars within the agricultural community. Gender studies in seed systems are limited, mainly focused on the role of women and capacity-building training for knowledge dissemination [16, 54, 55]. A few studies investigate the relevance of national seed policies in seed sector development, as well as the institutional opportunities and barriers to effective policy implementation [12, 56, 57]. The final theme of the studies centers on agrobiodiversity and conservation, particularly focusing on the role of seed systems in fostering landrace diversity [58], transitioning seed provisioning mechanisms [59], and assessing the impact of international gene banks on rice farming [32].

While these studies collectively offer insights into the complex dynamics of rice seed systems, encompassing aspects of seed quality, storage, policy impacts, farmer practices, and institutional frameworks across different geographic contexts, there is a limited exploration of farmers’ preferences regarding seed quality, packaging, and sources. Although some research touches upon factors influencing seed adoption and perceptions, there remains a gap in understanding the specific preferences of rice farmers in these key areas. Additionally, while studies examine the impact of seed enterprises, seed sources, and social capital on agricultural outcomes, there is a lack of focus on farmers’ preferences for seed quality attributes, packaging materials, and procurement sources. Given the crucial role of seed quality, packaging, and sources in ensuring crop productivity and resilience, a study focusing on rice farmers’ preferences in these aspects would provide valuable insights for policymakers, seed producers, and agricultural stakeholders. Moreover, addressing this gap is essential for developing targeted interventions and policies aimed at improving seed systems and enhancing agricultural sustainability in rice farming communities.

## 3. Materials and method

### 3.1 Study area and sample size

**Fig 2:**
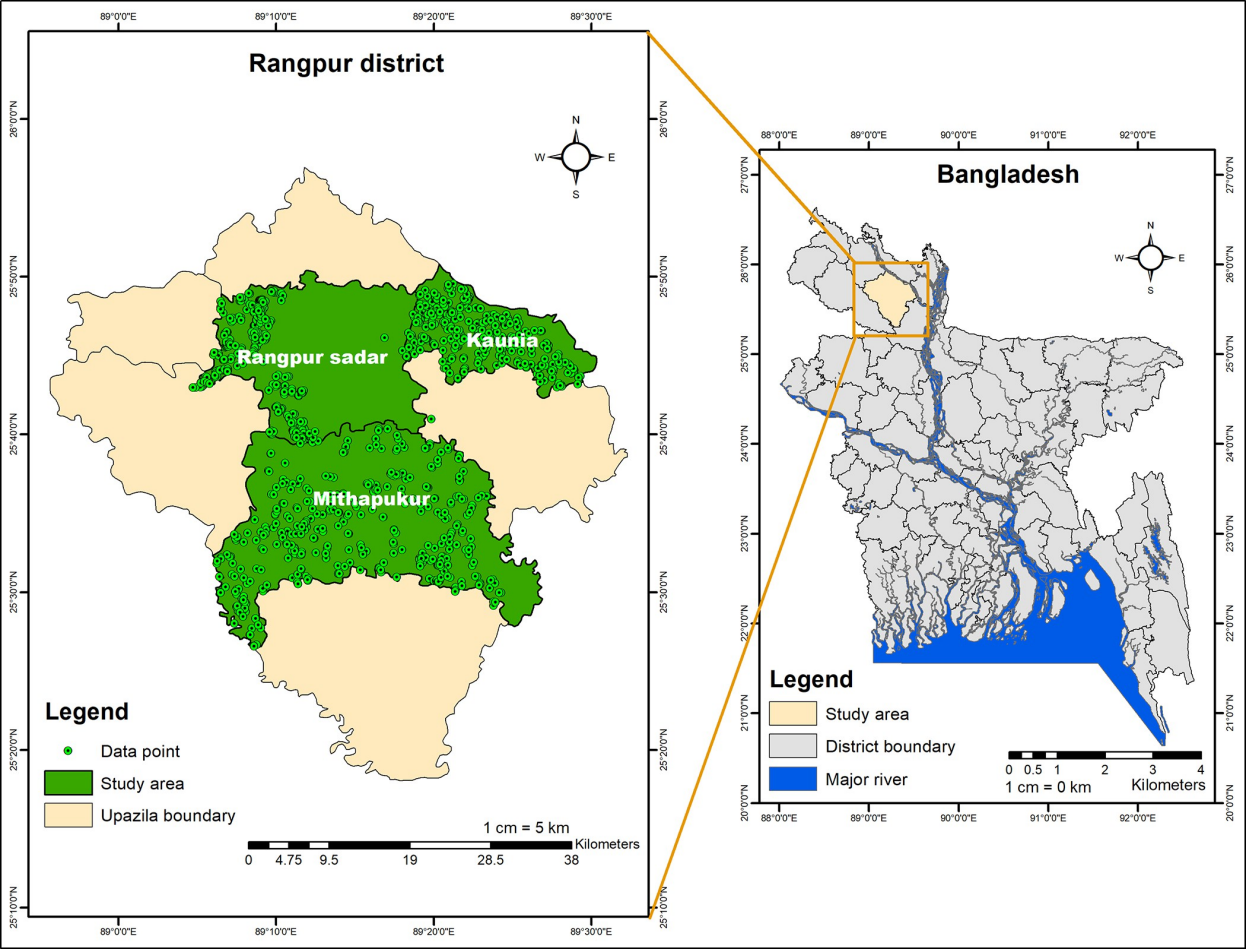
Study area map. GIS map prepared by the authors by using the administrative shapefile of Bangladesh. Shapefile republished from the Bangladesh Agricultural Research Council (BARC) database (http://maps.barcapps.gov.bd/index.php) under a CC BY license, with permission from Computer and GIS unit, BARC, original copyright 2014.

This research was carried out in the Rangpur district, situated in northern Bangladesh (Fig 2). This region is known for its biodiversity, fertile land, and high productivity, often referred to as the food basket of the country. However, the rice sector here is vulnerable to both natural and manmade disasters, leading to significant crop damage [1,8, 65]. Moreover, the rice seed system in this area is poorly established and faces numerous challenges. Farmers are increasingly adopting exotic rice seeds through cross-border seed exchange [2]. Community-based rice seed enterprises led by women are endeavoring to capitalize on the advantages of improved access to quality seeds, albeit to a limited extent [16]. Given these conditions, understanding rice farmers’ preferences is crucial for designing effective seed systems that meet local needs and promote sustainable agricultural development in northern Bangladesh. To achieve this, three distinct administrative sub-units (Upazilas) within the Rangpur district—Rangpur Sadar, Mithapukur, and Kaunia—were purposively selected for the study.

We opted to investigate rice-producing farmers for our research. This selection process aimed to prioritize an equal representation of farmers from all three Upazilas. The sample size of 1200 rice farmers was determined using a formula suggested by Kothari [66].

$$n=\frac{p*(1-z)*Z^{2}}{e^{2}}$$ (1)

Where n is the sample size. The estimated proportion of respondents (P) was set at 0.5 to maximize the number of participants. The standard error in the 95% confidence interval (Z) was 1.96, and the margin of error (e) that the researchers were willing to accept at 0.05. Therefore, the estimated sample size for a single Upazila was approximately 400, calculated as, $n=\frac{0.5*(1-0.5)*1.96^{2}}{0.05^{2}}$ = 385 ≈ 400.

However, due to incomplete data in four cases, a complete dataset of 1196 rice farmers were included in the analysis. The survey was conducted from April to June 2019. Face-to-face interviews were conducted using a structured questionnaire comprising both open-ended and closed-ended questions. The interviews were performed using the Computer Assisted Personal Interview (CAPI) method, with data collection facilitated by the KOBO software. Finally, the data were analyzed using StataMP 16.

### 3.2 Empirical settings

A theoretical framework was adopted based on the Expected Utility Theory (EUT) to guide the farmers’ preferences. According to the EUT, individuals make decisions by maximizing their expected utility derived from the perceived benefits and costs associated with different alternatives [67–69]. In this framework, the theory guides farmers’ decision-making process through decision-making criteria, utility maximization, trade-offs, determinants, and policy implications. The decision-making criteria include seed quality, packet types, packet sizes, and seed sources. By maximizing expected utility, farmers evaluate trade-offs and make choices based on their preferences for each criterion. The determinants of these choices include personal, social, and contextual factors. Finally, the framework will help to derive policy implications from the analysis to support sustainable agricultural practices and enhance farmers’ decision-making processes. So, to align these theoretical perspectives, for example, we can use econometric models, such as random utility models or discrete choice models, to estimate the expected utility of rice farmers of different seed quality levels, packet types, packet sizes, and seed sources.

#### 3.2.1 Ordered logit model

In this study, the variable ‘quality seed’ utilized by rice farmers was categorized as an ordered categorical variable with three levels: poor, average, and good. An ordered logit model was employed to examine the factors influencing farmers’ seed selections. Similarly, an ordered logit model was used to explore the influence of packet sizes (1 kg, 2 kg, 5 kg, and 10 kg) on rice farmers’ seed choices. The ordered logit model is a statistical technique that expands upon logistic regression. In this model, the ordinal variable Y is influenced by another variable Y*, which is continuous and unobserved. Y* is characterized by several threshold points, as shown by the following formula:

$$Y_{i}^{*}={\alpha_{0}+X}_{i}\beta +\varepsilon_{i}$$ (2)

Where, *X*_*i*_ is the vector of the explanatory variables (see Table 2); *α*_0_ is the intercept term; β is the vector of unknown parameters; and *ε*_*i*_ is a random error term assumed to have a zero mean and unit variance, and is normally distributed across all observations (i.e., standard normal distribution). The interpretation of ordered logit model coefficients can be challenging and sometimes misleading. Hence, we used marginal effects to provide a clearer and more coherent explanation of how changes in the independent variable affect the probabilities associated with the dependent variable. To address the issue of heteroskedasticity, we employed robust standard errors in our analysis. Furthermore, we assessed multicollinearity using the variance inflation factor (VIF) and found that the values for each variable were below five, suggesting no significant concern regarding multicollinearity in our dataset.

**Table 2:** Summary statistics of the respondents’ demographic and farm characteristics.

Particulars	Mean	STD	Min.	Max.	p-value
Age of farmers’ (years)	47.10	12.45	23.00	65.00	0.00
Farmers’ education (years)	6.83	4.94	0.00	18.00	0.00
Gender (%) Male Female	75.08 24.92	-	-	-	0.00
Operated land (decimal)	149.72	40.46	10.00	850.00	0.00
Farm category (%) Small Medium Large	75.33 18.06 6.61	-	-	-	0.00
Distance from home to seed dealers (km)	4.42	5.16	0.00	75.00	0.00
Distance from home to Upazila office (km)	19.27	8.92	1.00	64.00	0.00
Expected rice seed price (BDT/kg)	35.58	1.68	30.00	52.50	0.00
Rice seed sales price (BDT/kg)	44.44	4.15	32.00	70.00	0.00
Who advises using good seeds (%) Public dealer Private dealer Extension office Other farmers	20.32 11.12 12.37 56.19	-	-	-	0.00
Adulterated rice seed (Yes, %)	88.88	-	-	-	0.00
Seed is costly (Yes, %)	67.73	-	-	-	0.00
Pay more than fixed price (Yes, %)	13.71	-	-	-	0.00
Unavailability of desired seed (Yes, %)	7.36	-	-	-	0.00
Available but not in time (Yes, %)	11.37	-	-	-	0.00
Hassles to get rice seed (Yes, %)	6.77	-	-	-	0.00
Lack of knowledge about quality seed (Yes, %)	63.71	-	-	-	0.00
Dissatisfaction with the price paid (Yes, %)	76.09	-	-	-	0.00

Note: STD, Min., Max., and BDT denotes standard deviation, minimum, maximum, and Bangladeshi currency, respectively. The student’s t-test was employed.

#### 3.2.2 Multinomial logit (MNL) model

The ‘packet type’ of rice seed (*Polycoated* jute sack, plastic sack, and plastic packet) and ‘seed source’ (farm-saved, private, and public) were defined as an unordered categorical variable using the identification strategies. These were then used as the dependent variable in the MNL regression to explore the determinants of farmers’ preferences. The general form of the MNL regression is:

$$prob(Y_{i}=j)=\frac{e^{\beta_{i}x_{j}}}{1+\sum_{1}^{j}e^{\beta_{i}x_{j}}}$$ (3)

Eq (3) is normalized to eliminate uncertainty in the model. This normalization assumes that both β and the probabilities can be estimated as follow:

$$prob(Y_{i}=0)=\frac{1}{1+\sum_{1}^{j}e^{\beta_{i}x_{j}}}$$ (4)

Where for the i^th^ farmer, *Y*_*i*_ is the observed outcome and *x*_*i*_ is the explanatory variable. *β*_*i*_ is the corresponding coefficient for each explanatory variable. The explanatory variables included in the empirical models are presented in Table 2. We used the marginal effect to interpret the effect of independent variables on dependent variable.

#### 3.2.3 Propensity score matching (PSM)

We used PSM by Rosenbaum and Rubin [70] to overcome selection bias and to estimate the overall impact of good quality seed and formal seed sources on the rice yield. The functional form of PSM is as follows:

p(x)=Pr(1=1|X=x) (5)

Where, x represents a vector of observed covariates, such as demographic characteristics or pre-treatment variables. Under conditional independence, the average treatment on treated (ATT) for rice yield is computed as:

$$ATT=E(Y_{1}-Y_{0}|X,I=1)$$ (6)

In this study, we used Nearest Neighbour (NN), Kernel, Radius, and Stratification matching methods to estimate ATT.

The functional form of the **Nearest Neighbour (NN)** matching method can be written as:

$${NM}^{M}=\frac{1}{N^{T}}{\Sigma Y}_{i}^{T}-\frac{1}{N^{T}}\Sigma w_{j}Y_{j}^{c}$$ (7)

Where, T is the set of treated, and C is the set of control units and $Y_{i}^{T}$ and $Y_{j}^{c}$ is the observed outcomes of the treated and control units, and the weights *w*_*j*_ are defined as, *w*_*j*_ = Σ*w*_*ij*_.

The **kernel matching** method, as outlined by Heckman et al. [71], can be summarized as follows:

$$E(\Delta Y)=\frac{1}{N}\sum_{i\in T}\left[Y_{i,1}-\frac{\sum_{j=1}^{N_{i}^{c}}Y_{j,0}^{i}K(\frac{P(X_{j,0}^{i})-P(X_{i,1})}{b_{w}}}{\sum_{j=1}^{N_{i}^{c}}K(\frac{P(X_{j,0}^{i})-P(X_{i,1})}{b_{w}}}\right]$$ (8)

Where, T represents the set of observations in the treatment group, specifically the adopters, and N denotes the total number of treated cases; *Y*_*i*,1_ and *X*_*i*,1_ are the dependent and independent variables for the *i*^*th*^ treated case; $Y_{j,0}^{i}$ and $X_{j,0}^{i}$ are the dependent and independent variables for the *j*^*th*^ control case that is within the neighborhood of treatment case *i*, i.e. for which $\left|P(X_{j,0}^{i})-P(X_{i,1})\right|$ < *b*_*w*_/2; $N_{i}^{c}$ is the number of comparison cases within the neighborhood of *i*; *K(•)* is a kernel function; and *b*_*w*_ is a bandwidth parameter.

Functionally, the **radius matching** method is as follows:

$$R^{M}=\frac{1}{N^{T}}{\Sigma Y}_{i}^{T}-\frac{1}{N^{T}}\Sigma w_{j}Y_{j}^{c}$$ (9)

Where, the weights *w*_*j*_ are defined as, *w*_*j*_ = Σ*w*_*ij*_.

The study samples were grouped or strata based on estimated propensity scores in **stratification matching**. As per the design of this procedure, each block formed ensures a balance in covariates, and the assignment of treatment can be deemed random. Therefore, when considering the blocks defined by intervals of the propensity score, denoted by q, the program calculates the following within each block.

$$\tau_{q}^{S}=\frac{\Sigma_{i\in I(q)}Y_{i}^{T}}{N_{q}^{T}}-\frac{\Sigma_{j\in I(q)}Y_{j}^{C}}{N_{q}^{C}}$$ (10)

Where I(q) is the set of units in block q; while $N_{q}^{T}$ and $N_{q}^{C}$ are the numbers of treated and control units in block q.

The estimator of ATT using the stratification method is calculated as follows:

$$\tau^{S}=\Sigma_{q=1}^{Q}\tau_{q}^{S}\frac{\Sigma_{i\in I(q)}D_{i}}{\Sigma_{\forall i}D_{i}}$$ (11)

Where the corresponding fraction of treated units gives the weight for each block, and Q is the number of blocks, D = {0, 1} is the indicator of exposure to treatment.

#### 3.2.4 Nature of independent variables

The independent variables used in the models include socio-demographic variables (such as farmers’ age, education level, and gender); farm characteristics (e.g., farm type, land ownership, yield, and area cultivated); infrastructure (e.g., the distance to seed dealers and Upazila office); access to information (e.g., advice from public and private dealers, extension office, and other farmers); seed quantity and quality (e.g., seed quantity used, adulterated seed, unavailability of desired seed, hassles to get seed, and lack of knowledge about quality seed); and seed price-related variables (e.g., farmers expected price, high seed cost, and dissatisfaction with the price paid). Summary statistics of these variables are presented in Table 2.

### 3.3 Ethical considerations

Ethical considerations were meticulously addressed in this study. Before participation, informed written consent was obtained from all individuals, ensuring they were fully informed about the study’s purpose and potential risks. Confidentiality and anonymity measures were implemented to safeguard the privacy of participants. It is important to note that this study adhered to local legislation and institutional guidelines, obviating the need for ethical approval. Furthermore, all procedures conducted in this study followed the principles outlined in the Helsinki Declaration regarding the involvement of human subjects.

## 4. Results

### 4.1 Demographic and farm characteristics of the sample farmers

Table 2 presents a concise overview of the demographic and farm characteristics of the respondents. The average age of the rice farmers was 47.10 years, with an average educational attainment of 6.83 years. The male population constituted a dominant majority, with 75.08% of the respondents being male. On average, the operated land size was 149.72 decimals. In terms of farm categorization, the majority of the farms were in a small category, accounting for 75.33% of the sample, followed by medium-sized farms (18.06%) and large farms (6.61%).

An interesting finding was that 56.19% of the farmers sought advice on using good seeds from other farmers, followed by public dealers, extension offices, and private dealers. Farmers highlighted various challenges and issues related to seed quality and systems. The most commonly reported concern was the presence of adulterated rice seeds and high seed costs were a significant issue for 88.88% and 67.73% of the farmers, respectively. About 76.09% of farmers expressed dissatisfaction with the price they paid for rice seeds. Furthermore, a notable proportion of farmers faced difficulties such as the unavailability of desired seeds, seeds not being available in time, hassles in obtaining seeds, and a lack of knowledge about quality seeds.

### 4.2 Heterogeneity of quality seed, packet type, packet size, and seed source

Quality seeds ensure better rice yield. In the surveyed regions (Table 3), approximately 51.76% of farmers used good quality seeds, while only 17.81% opted for low-quality seeds. Approximately 56.05% of small farmers utilized good quality seeds acquired from public or private sources, followed by medium and large farmers. Small farmers often faced seed preservation and storage challenges due to limited experience and capacity. Typically, they sold their produce for immediate consumption expenditure and rarely retained rice seeds for future seasons. In contrast, the majority of large-scale farmers relied on average-quality seeds primarily sourced from their own seed stock and private dealers. These were the farmers in the study areas who possessed the capability to produce, store, and maintain seeds for the next season.

**Table 3:** Rice farmers’ preferences for quality seed, packet type, packet size, and seed sources by farm category in frequencies.

Particulars	Farm category				Pearson Chi^2^ test
	Small	Medium	Large	All	
**Seed quality**					
Poor	157 (17.43)	37 (17.13)	19 (24.05)	213 (17.81)	34.99***
Average	239 (26.52)	89 (41.20)	36 (45.57)	364 (30.43)	
Good	505 (56.05)	90 (41.67)	24 (30.38)	619 (51.76)	
**Packet type**					
*Polycoated* jute sack	732 (81.24)	167 (77.31)	57 (72.16)	956 (79.93)	11.09**
Plastic sack	82 (9.10)	27 (12.50)	16 (20.25)	125 (10.45)	
Plastic packet	87 (9.66)	22 (10.19)	6 (7.59)	115 (9.62)	
**Packet size**					
1 kg	39 (4.33)	10 (4.63)	4 (5.06)	53 (4.43)	18.50***
2 kg	167 (18.54)	28 (12.96)	5 (6.33)	200 (16.73)	
5 kg	427 (47.39)	127 (58.80)	39 (49.37)	593 (49.58)	
10 kg	268 (29.74)	51 (23.61)	31 (39.24)	350 (29.26)	
**Seed source**					
Farm-saved	274 (30.42)	97 (44.91)	29 (36.71)	400 (33.45)	29.40***
Private	288 (31.96)	69 (31.94)	34 (43.04)	391 (32.69)	
Public	339 (37.62)	50 (23.15)	16 (20.25)	405 (33.86)	
Total observations	901	216	79	1196	-

Figures in the parentheses indicate percentages of farm category. Significance level

*** p<0.01

** p<0.05

* p<0.10

For packet type, *polycoated* jute sacks were the most preferred option among farmers. The *polycoated* sacks offer farmers a durable and effective solution for rice seed storage, ensuring better preservation and protection of seeds during transportation and storage. In terms of packet size, the majority of farmers preferred 5 kg packets. The 10 kg packets were also popular among small, medium, and large farmers, while the 1 kg packets were less frequently chosen by all farmers. According to respondents, 5–10 kg packets were more cost-effective and convenient to store and handle. The preferences for seed sources showed minimal variation on average, but significant (p<0.01) differences were observed across different categories of farmers. Among these categories, most small farmers relied on rice seeds from public sources (37.62% responses), medium farmers predominantly used their own stock as a seed source (44.91%), and large farmers primarily relied on private sources for seeds (43.04%).

There was a significant association (p<0.01) between seed quality and yield advantages of rice (Table 4). The good quality seeds resulted in higher yields compared to poor or average quality seeds. For example, in the small farm category, the yield advantage of good quality seeds compared to poor quality seeds was 19.81%. This percentage was even higher in the medium and large farm categories, with advantages of 26.05% and 28.50%, respectively. In terms of packet type, *polycoated* jute sacks were associated with higher yields compared to plastic sacks and plastic packets across all farm categories. Table 4 shows that different packet sizes had varying effects on yield and effects were found to be statistically significant (p<0.10). In general, 2 kg packets consistently resulted in higher yields than other sizes, similar for 5 kg package. For large farmers, however, increasing the size of the packets increased yield except 10 kg package. Overall, we observed a farmers employing 2–5 kg seed packages follow seed recommendation rate resulting better yield. significant (p<0.10) variation in yields across farm categories when employing different sizes of seed packets. Regarding seed sources (p<0.05), there were variations in yields across farm categories. We found that private seed sources consistently resulted in higher yield advantages compared to public and farmers’ own sources because of hybrid rice varieties’ dominance. Notably, large-scale farmers using public source seeds achieved higher yields in comparison to small and medium-scale farmers. It was important to consider that farmers’ own preserved seeds did not undergo quality maintenance procedures that could potentially result in lower yields (ranging from 0.96 t/ha to 1.01 t/ha) compared to formal sources. This suggested that the self-preserved seeds used by farmers may not be as optimized for yield maximization as the seeds obtained from formal public and private sources.

**Table 4:** Farmers’ yield advantages of rice seed (ton/ha) by quality, packet type, packet size, and sources across farm categories.

Particulars	Farm category				F-test
	Small	Medium	Large	All	
**Seed quality**					
Poor	4.19 (0.46)	4.03 (0.41)	4.07 (0.36)	4.09 (0.45)	3.53***
Average	4.42 (0.57)	4.41 (0.37)	4.48 (0.54)	4.39 (0.53)	
Good	5.02 (0.62)	5.08 (0.46)	5.23 (0.59)	5.03 (0.60)	
Yield advantage of good quality seed compared to poor (%)	19.81	26.05	28.50	22.98	-
**Packet type**					
*Polycoated* jute sack	5.02 (0.60)	5.08 (0.43)	5.25 (0.44)	5.09 (0.57)	3.32***
Plastic sack	4.91 (0.42)	5.04 (0.35)	5.14 (0.56)	4.99 (0.42)	
Plastic packet	4.80 (0.49)	4.81 (0.30)	4.86 (0.31)	4.81 (0.43)	
**Packet size**					
1 kg	4.32 (0.24)	4.87 (0.46)	4.93 (0.30)	4.69 (0.30)	1.88*
2 kg	5.07 (0.24)	5.08 (0.55)	5.12 (0.26)	5.07 (0.52)	
5 kg	4.59 (0.53)	5.06 (0.41)	5.14 (0.42)	4.91(0.49)	
10 kg	4.54 (0.70)	5.03 (0.35)	5.05 (0.67)	4.86 (0.68)	
**Seed source**					
Farm-saved	3.98 (0.49)	4.03 (0.43)	4.08 (0.43)	4.01 (0.48)	2.30**
Private	5.03 (0.56)	5.01 (0.40)	5.02 (0.38)	5.02 (0.52)	
Public	4.96 (0.66)	4.93 (0.80)	5.09 (0.42)	4.97 (0.64)	
Total observations	901	216	79	1196	-

Figures in the parentheses indicate standard deviations. Significance level

*** p<0.01

** p<0.05

* p<0.10

We observed significant (p<0.05) price variations of poor, average, and good quality rice seeds across farm categories (Table 5). On average, the cost of good quality seeds ranged between 44.57 and 45.48 BDT/kg. The average price differential between good and poor-quality seeds was 9.04%. Small farmers paid significantly less than large farmers for good quality seeds, indicating that they had little bargaining power and good social relations with seed dealers. The *polycoated* jute sacks had the highest mean sales price across all farm categories, with large farms commanding the highest price of 48.93 BDT/kg. Plastic sacks and plastic packets had lower mean sales prices, with plastic packets being the least expensive option. This indicated that the type of packaging material (p<0.05) could play a crucial role in determining the price of seeds.

**Table 5:** Sales price of rice seed (BDT/kg) by quality, packet type, packet size, and sources across farm categories.

Particulars	Farm category				F-test
	Small	Medium	Large	All	
**Seed quality**					
Poor	40.87 (3.91)	41.23 (4.06)	42.05 (5.80)	41.28 (4.61)	2.08**
Average	42.43 (3.77)	42.58 (4.35)	43.09 (3.55)	42.69 (4.09)	
Good	44.57 (4.26)	45.04 (5.02)	45.48 (3.46)	45.01 (4.02)	
Price of good quality seed compared to poor (%)	9.05	9.24	8.16	9.04	-
**Packet type**					
*Polycoated* jute sack	45.16 (4.37)	46.21 (4.47)	48.93 (6.39)	46.77 (3.89)	9.29***
Plastic sack	43.78 (1.98)	44.11 (4.26)	45.10 (4.28)	44.30 (5.06)	
Plastic packet	41.65 (4.04)	42.11 (4.26)	43.01 (3.60)	42.06 (4.35)	
**Packet size**					
1 kg	44.23 (3.83)	45.07 (4.04)	44.92 (3.48)	44.68 (4.75)	1.81*
2 kg	44.23 (3.85)	44.69 (5.08)	44.09 (4.77)	44.32 (4.12)	
5 kg	45.63 (1.25)	45.87 (5.25)	46.45 (4.28)	45.91 (3.87)	
10 kg	43.67 (4.12)	44.05 (4.10)	45.15 (4.07)	44.27 (4.10)	
**Seed source**					
Farm-saved	44.59 (3.85)	45.24 (4.68)	46.27 (3.90)	45.28 (4.10)	2.07**
Private	44.46 (4.27)	44.10 (5.07)	45.00 (3.72)	44.51 (4.28)	
Public	44.05 (3.97)	43.93 (4.56)	43.40 (2.29)	43.76 (4.06)	
Total observations	901	216	79	1196	-

Figures in the parentheses indicate standard deviations. Significance level

*** p<0.01

** p<0.05

* p<0.10

Among all farm categories, the 5 kg packet size exhibited slightly higher sales in comparison to other sizes (p<0.10). This can be attributed to the increased market demand for this specific size in the study areas. Secondly, considering farm categories, there seemed to be minimal variation in sales prices based on packet sizes. Small, medium, and large farms generally had similar sales prices for each packet size. The source of the rice seed (p<0.05) significantly affected its pricing. Farmers’ own seeds had slightly higher sales prices across all farm categories, ranging from 44.59 BDT/kg for small farmers to 46.27 BDT/kg for large farmers. This is because of the associated storage cost and the availability of desired seed varieties. Public seed sources had the lowest sales prices since they were subsidized by the governments.

Table 6 reveals that across all farm categories, good quality seed led to a higher expected price compared to poor quality seed (p<0.01). The average price differences between poor and good quality rice seeds ranged from 12.82% to 14.77%, with the highest difference observed in the small farm category. This suggests that farmers were willing to pay a premium for better quality seeds, recognizing their potential for improved yields and efficiency. Nevertheless, the actual sales price remained approximately 11% higher than the farmers’ anticipated price for good quality seeds. The type of packet used for seed packaging also influences the willingness to pay price (p<0.01). *Polycoated* jute sacks had the highest prices among the three packet types for all farm categories. This suggests that farmers place a higher value on rice seeds packaged in *polycoated* jute sacks, possibly due to perceived advantages such as better protection or durability.

**Table 6:** Farmers’ expected rice seed price (BDT/kg) by quality, packet type, packet size, and sources across farm categories.

Particulars	Farm category				F-test
	Small	Medium	Large	All	
**Seed quality**					
Poor	35.41 (1.30)	35.30 (1.22)	36.36 (3.20)	35.58 (1.56)	3.33***
Average	36.51 (1.63)	36.91 (2.01)	37.40 (0.98)	36.90 (1.68)	
Good	40.64 (1.53)	40.46 (1.15)	41.02 (4.62)	40.67 (1.72)	
Price of good quality seed compared to poor (%)	14.77	14.62	12.82	14.31	-
**Packet type**					
*Polycoated* jute sack	38.51 (1.45)	39.35 (3.21)	40.72 (2.95)	39.51 (2.00)	9.88***
Plastic sack	36.35 (1.65)	36.48 (0.90)	37.55 (3.48)	36.76 (1.89)	
Plastic packet	35.48 (1.47)	35.20 (1.09)	35.94 (3.03)	35.51 (1.56)	
**Packet size**					
1 kg	35.90 (1.88)	35.40 (1.09)	35.18 (1.02)	35.47 (2.13)	8.46***
2 kg	35.38 (1.33)	35.70 (1.67)	37.01 (4.18)	36.01 (1.28)	
5 kg	36.41 (1.23)	37.28 (4.60)	39.10 (5.63)	37.54 (2.67)	
10 kg	35.30 (1.21)	35.28 (1.14)	35.23 (0.87)	35.25 (1.26)	
**Seed source**					
Farm-saved	35.34 (1.17)	35.60 (2.02)	35.65 (2.58)	35.51 (1.55)	5.01***
Private	35.66 (1.37)	35.50 (1.17)	35.75 (1.91)	35.60 (1.39)	
Public	35.67 (1.84)	36.11 (0.85)	37.77 (5.07)	36.48 (1.68)	
Total observations	901	216	79	1196	-

The figure in the parentheses indicates standard deviations. Significance level

*** p<0.01

** p<0.05

* p<0.10

The Table 6 highlights that larger packet sizes generally corresponded to higher expected prices of seeds (except 10 kg), potentially reflecting economies of scale or higher demand for larger quantities of rice seeds (p<0.01). Across all farm categories, it was evident that farmers highly preferred 5 kg size packets, as indicated by their willingness to pay a higher price than for other packet sizes. However, farmers’ own seeds and private dealers’ seeds were perceived to have relatively consistent prices (p<0.01). Public seed sources exhibited slight variations in prices, with larger farms potentially showing a greater willingness to pay. In sum, the higher expected prices of rice seeds from public sources indicate that farmers in the study areas prioritized public seed sources.

### 4.3 Determinants of rice farmers’ preferences for quality seed, packet size, packet type, and seed source

The parameter estimates and average marginal effects derived from the ordered logit regression for quality seed and packet size, as well as the multinomial logit regression for packet types and seed sources, are provided in Table 7 and S1 Table, respectively.

**Table 7:** The average marginal effects of the estimated coefficient of the ordered logit model for quality seed and packet size, and the multinomial logit model for packet types and seed sources.

Parameters	Ordered logit regression							Multinomial logit regression					
	Quality seed			Packet sizes				Packet types			Seed sources		
	Poor	Average	Good	1 kg	2 kg	5 kg	10 kg	*Polycoated* jute sack	Plastic sack	Plastic packet	Farm-saved	Private	Public
Farmers’ age (years)	0.002	0.004	-0.006	-0.001*	-0.005*	-0.002*	0.009*	-0.008	-0.003	0.005	0.005	0.0003	-0.003
Farmers’ age squared (years)	-0.0001	-0.0001	0.0002	0.0001	0.0001	0.0002	-0.0001	0.002*	0.0001	-0.0001	-0.0001	-0.0002	0.0001
Farmers’ education (years)	-0.0002	-0.001	0.001	-0.0001	-0.0002	-0.0001	0.001	0.004*	-0.004***	-0.0002	-0.006*	0.0002*	0.008*
Gender (1 = male)	0.021*	0.046*	-0.067*	-0.008**	-0.036**	-0.023*	0.066**	-0.058***	0.035***	0.023	0.081	0.0004	-0.086
Small farms (ref. large)	-0.146***	-0.172***	0.318***	0.010	0.043	0.029	-0.083	0.165*	-0.103*	-0.062*	0.307**	-0.017***	0.323**
Medium farms (ref. large)	-0.050***	-0.124**	0.174**	0.013	0.054	0.011**	-0.078	0.058	-0.040***	-0.018**	-0.252	-0.005***	0.287*
Operated land in decimal (logarithm)	0.019*	0.038*	-0.057*	-0.0001	-0.001	-0.001	0.002	0.009	0.0004	-0.009	0.018	-0.001	0.023
Distance from home to seed dealers (km)	0.002**	0.004**	-0.005**	-0.001***	-0.004***	-0.002***	0.006***	-0.002***	-0.0003	0.003***	0.003*	-0.002**	-0.001*
Distance from home to Upazila office (km)	0.001	0.001	-0.002	0.001**	0.002**	0.001**	-0.004**	-0.006***	0.003*	0.003***	0.005	-0.0001	-0.006
Access to public seed sources (ref. farm-saved)	-0.102***	-0.235***	0.337***	-0.020**	-0.086***	-0.062*	0.168**	0.026**	-0.033**	-0.059**	-	-	-
Access to private seed sources (ref. farm-saved)	-0.006	-0.012	0.018	-0.012***	-0.054***	-0.034***	0.100***	-0.006	0.002*	-0.009	-	-	-
Advice from public dealers (ref. other farmers’)	-0.090***	-0.125***	0.215***	-0.031***	-0.116***	-0.001	0.148***	0.030	-0.016	-0.014	-0.131**	-0.0002	0.143***
Advice from private dealers (ref. other farmers’)	-0.301***	-0.127***	0.428***	-0.028***	-0.105***	-0.006	0.128***	0.044	0.047*	-0.004	-0.534***	0.004**	0.548***
Advice from extension office (ref. other farmers’)	-0.118***	-0.133***	0.250***	-0.024***	-0.092***	-0.001	0.117***	0.052	0.067***	-0.016**	-0.081**	0.002	0.085*
Adulterated seed (1 = yes)	0.400***	0.085**	-0.485***	0.006**	0.025**	0.017**	-0.048**	-0.007**	-0.015*	0.022**	0.053**	-0.002	-0.064**
Unavailability of desired seed (1 = yes)	0.078*	0.102***	-0.180***	-0.014***	-0.067***	0.077**	0.158***	0.005	0.030	-0.025	0.035**	-0.002*	-0.045**
Hassles to get seed (1 = yes)	0.045**	0.071**	-0.116**	-0.015*	-0.057*	0.007	0.078*	-0.085*	-0.013	0.072	0.275**	0.006*	-0.281**
Available but not in time (1 = yes)	0.005*	0.011*	-0.016*	-0.003	-0.014	-0.008	0.025	-0.046	0.029	0.017	0.023	-0.002	-0.035
Lack of knowledge about quality seed (1 = yes)	0.057***	0.125***	-0.181***	0.004	0.015	0.008	-0.027	-0.067*	-0.010	0.057**	0.244	0.003	-0.267
Seed is costly (1 = yes)	0.071**	0.118***	-0.189**	-0.0002	-0.001	-0.0001	0.001	-0.046**	-0.015**	0.061**	0.466***	-0.513***	-0.979***
Pay more than the fixed price (1 = yes)	0.050**	0.080***	-0.130***	-0.002	-0.010	-0.005	0.017	-0.035	0.016	0.020	0.066	-0.001	-0.089
Dissatisfaction with the price paid (1 = yes)	0.102***	0.269***	-0.371***	0.002	0.009	0.005	-0.016	0.036	0.005	-0.031	0.335**	-0.002	-0.367**
Rice seed sales price (BDT/kg)	0.005	0.011	-0.016	0.001	0.003	0.002	-0.006	-0.026***	-0.005**	0.021***	0.052*	-0.0001	-0.042*
Price difference between expected and sales prices of rice (BDT/kg)	0.003	0.006	-0.009	0.001	0.005	0.003	-0.009	-0.021***	-0.003**	0.018***	0.038***	-0.0002	-0.058***
Quantity of rice seed used in hectares (logarithm)	0.005***	0.009***	-0.014***	-0.001	-0.002	-0.001	0.004	0.002	-0.001	-0.001	0.009**	-0.0002***	-0.019**
Squared quantity of rice seed used in hectares (logarithm)	0.0001*	0.0002*	-0.0001*	-0.0002	-0.0002	-0.0001	0.0002	0.0004	-0.0002	0.0002	0.0001*	-0.0002***	-0.0003
Rice yield (ton/ha)	-0.020*	-0.041*	0.060*	-	-	-	-	0.060**	-0.041*	-0.018**	-0.032	0.0004	0.061
*Polycoated* jute sack (ref. plastic packet)	-	-	-	-0.081***	-0.241***	0.074**	0.249***	-	-	-	-0.253*	-0.002	0.285*
Plastic sack (ref. plastic packet)	-	-	-	-0.016***	-0.074***	-0.086**	0.177***	-	-	-	0.204**	0.001	-0.216*
1 kg packet seed (ref. 10 kg)	-	-	-	-	-	-	-	-0.489***	-0.047**	0.442***	0.191**	-0.003***	-0.188**
2 kg packet seed (ref. 10 kg)	-	-	-	-	-	-	-	-0.321***	0.149***	0.172***	0.270***	-0.002***	-0.268**
5 kg packet seed (ref. 10 kg)	-	-	-	-	-	-	-	-0.097**	0.047***	0.051**	0.048	-0.003***	-0.045
Good quality seed (ref. poor)	-	-	-	-	-	-	-	-	-	-	-0.446***	0.003*	0.459***
Average quality seed (ref. poor)	-	-	-	-	-	-	-	-	-	-	-0.156	0.003**	0.169

Significance level

*** p<0.01

** p<0.05

* p<0.10

#### 4.3.1 Quality seed

The gender variable (p<0.10) had a negative impact on the selection of good quality rice seed, indicating that male farmers were more inclined to choose lower quality seed compared to their female counterparts. Small (p<0.01) and medium (p<0.05) farmers were less likely to opt for poor and average quality seeds but showed a higher likelihood of selecting good quality seeds compared to large farmers. Increasing land size (p<0.10) resulted in a significant decrease in the utilization of good quality seed. The preference for good quality seed decreased as the distance between farmers’ residences and seed dealers increased (p<0.05). Access to public seed sources, as well as receiving advice from public and private dealers and extension offices, had a positive marginal effect on the selection of good quality seed. These effects were statistically significant at a 1% probability level, highlighting the influence of formal seed sources and advisory services on farmers’ preference for adopting good quality seeds. The rice yield (p<0.10) positively influenced the choice of good quality seed, indicating that farmers with higher yields were more inclined to select such seeds. Farmers who used a higher quantity of seed per hectare of land (p<0.01) tended to prefer poor seed quality. Additionally, the presence of adulterated seed (p<0.01), perceiving seed as expensive (p<0.05), unavailability of desired seed (p<0.01), paying more than the fixed price (p<0.01), availability but not timely access (p<0.10), dissatisfaction with the price paid (p<0.01), lack of knowledge (p<0.01), and facing difficulties in obtaining seed (p<0.05) all significantly reduced farmers’ inclination to choose the good quality rice seed.

#### 4.3.2 Packet sizes

Farmers’ age (p<0.10) was negatively associated with choosing smaller packet sizes (1 kg, 2 kg, and 5 kg) but positively related to choosing a larger packet size (10 kg), indicating that younger farmers are more likely to prefer smaller packet sizes. Male farmers (p<0.05) were more likely to choose larger packet sizes than female farmers due to economies of scale of production. As the distance from farmers’ residences to seed dealers (p<0.01) increased, they preferred to purchase 10 kg packets, while the opposite was true for distances to Upazila (p<0.05). The reason was cost-effectiveness, transportation, and storage considerations, reducing seed purchase frequency.

Having access to public (p<0.05) and private (p<0.01) seed sources, as well as receiving advice from the public (p<0.01) and private (p<0.01) dealers and extension offices (p<0.01), had a negative effect on choosing smaller packet sizes (1 kg, 2 kg, and 5 kg) but a positive effect on choosing a larger packet size (10 kg), indicating that farmers with access to formal seed sources and advisory services were more likely to choose larger packet sizes. The presence of adulterated seeds (p<0.05) increased rice farmers’ preference to buy smaller packet sizes (1 kg, 2 kg, and 5 kg). Moreover, the lack of availability of desired seeds (p<0.01) and the difficulties faced in obtaining them (p<0.10) compelled farmers to opt for purchasing seeds in 5 kg or 10 kg packet sizes. The utilization of *polycoated* jute sacks had significant positive effects on the choice of 5 kg (p<0.05) and 10 kg (p<0.01) packet sizes, while the plastic sacks had significant positive effects on the choice of 10 kg packet sizes (p<0.01) only compared to plastic packets.

#### 4.3.3 Packet types

As farmers’ age increased, their preference for *polycoated* jute sacks showed a positive trend at a 10% significance level, suggesting that older farmers were more inclined than younger farmers to pay higher prices for *polycoated* jute sacks of rice seed. This could be attributed to the accumulated experience and knowledge of older farmers in agricultural practices, leading them to develop a preference for jute sacks based on factors such as familiarity, trust in the retention of seed quality, and perceived durability. Education increased the choice for *polycoated* jute sacks (p<0.10) and decreased the preference for plastic sacks (p<0.01) compared to plastic packets. The male farmers prioritized plastic sacks (p<0.01), whereas female farmers prioritized *polycoated* jute sacks (p<0.01). Compared to large farmers, small and medium farmers preferred *polycoated* jute sacks over plastic sacks and plastic packets.

Farmers’ distance from seed dealers and the Upazila office decreased their likelihood of choosing *polycoated* jute sacks and increased their likelihood of choosing plastic packets at a 1% significance level. The availability of public seed sources (p<0.05) significantly increased the likelihood of choosing *polycoated* jute sacks, while the availability of private seed sources (p<0.10) significantly increased the likelihood of choosing plastic sacks. Similarly, the advice from private dealers (p<0.10) and the extension office (p<0.01) increased farmers’ preference for adopting plastic sacks.

Adulterated seed, high seed cost, hassles to get seed, and lack of knowledge about quality seed significantly decreased farmers’ preference for selecting *polycoated* jute sacks and plastic sacks. The sales price of seeds and the price difference between expected and actual prices had a significant negative effect on the likelihood of choosing *polycoated* jute sacks (p<0.01) and plastic sacks (p<0.05), while it had a significant positive effect on the likelihood of choosing plastic packets (p<0.01). That means higher prices and larger price discrepancies discourage the choice of certain types of seed packaging materials, as farmers perceive them to be less affordable or cost-ineffective, leading to expected strategic behavior. The preference of farmers for *polycoated* jute sacks (p<0.05) associated with higher yield suggested that farmers perceived *polycoated* jute sacks as more traditional, reliable, and associated with higher-quality produce. Our findings also indicated that farmers tended to prefer *polycoated* jute sacks as the demand for packet size increased, particularly for 10 kg packets. Conversely, plastic sacks were preferred for 2 kg and 5 kg size packets at a 1% significance level. So, the farmers’ preferences for larger quantities in *polycoated* jute sacks can be attributed to the potential for a more cost-effective choice.

#### 4.3.4 Seed sources

Farmers’ level of education significantly decreased the probability of choosing farm-saved seeds (p<0.10). Educated farmers were seen to choose public and private seeds over their own. Small farmers demonstrated a higher likelihood of selecting farm-saved seed (p<0.05) and public seed (p<0.05), whereas medium-sized farmers exhibited a higher preference for public seed (p<0.01) in comparison to large farmers. The distance from home to seed dealers had a significant negative effect on the probability of choosing private (p<0.05) and public (p<0.10) seed sources but increased the preference to use their own seeds (p<0.10). That means, farmers were more likely to opt for their own seeds as the distance increased. Farmers who received advice from formal seed systems dealers were more likely to choose private or public seeds. Similarly, the advice provided by extension officers (p<0.10) influenced farmers’ decisions to opt for formal seed sources.

The factors, adulterated seed (p<0.05) and unavailability of desired seed (p<0.05) had reduced the probability of choosing private or public sources over farm-saved seed sources. Additionally, farmers who encountered hassles in obtaining seed (p<0.05) or were dissatisfied with the price paid (p<0.05) were more inclined to choose their own seed but less likely to select public seed sources. As anticipated, expensive seeds (p<0.10) and experiencing a price difference between expected and sales prices (p<0.01) reduced rice farmers’ preference for accessing public seed sources. Farmers who used a higher (overdose) quantity of rice seed per hectare of land (p<0.05) tended to prefer farm-saved seed sources as they got adequate quantities and desired varieties of seeds. This suggests that the practices of recommended seed rates could affect farmers’ decisions about accessing public or private seed sources. Farmers who used *polycoated* jute sacks (p<0.10) were more likely to rely on public seed sources. On the contrary, farmers who used plastic sacks (p<0.10) were less likely to rely on public seed sources. Farmers’ preferences for smaller seed packets (i.e., 1 kg, 2 kg, and 5 kg) increased for their use of own seed but decreased for public and private seed sources. Farmers who prioritized good quality seed were more inclined to choose public (p<0.01) and private (p<0.10) seed sources. This could be due to the perception that public and private seed sources are more reliable or regulated in terms of quality standards.

According to the pseudo R-squared and Wald chi-square statistics (S1 Table), the model was well-fitted, and the included parameters had a statistically significant effect on the outcome variable, resulting in a perfect prediction.

### 4.4 Impact of good quality and formal seed sources on rice yield

The impact of good quality seed usage and access to formal seed sources on the rice yield was estimated using the propensity score matching method (Table 8). A common support region was selected to ensure the method’s validity, and the balancing property was satisfied. As previously mentioned, using good quality seed and increased access to formal seed sources enhanced rice productivity compared to poor quality and informal sources of seed. Nonparametric matching estimates generated by various matching methods revealed that farmers who used good quality seed experienced an average increase in rice yields ranging from 0.07 t/ha to 0.28 t/ha (p<0.01), significantly higher than the yields of poor-quality seed users in the study areas. When considering different farm categories and matching algorithms, small farmers who employed good quality seed observed average yield increases ranging from 0.09 t/ha to 0.17 t/ha (p<0.05), medium farmers experienced increases ranging from 0.04 t/ha to 0.07 t/ha (p<0.10), and large farmers witnessed increases ranging from 0.06 t/ha (p<0.10) to 0.18 t/ha (p<0.01). Here, the variation in yield advantages across farm categories was explained to the efficiency of farmers.

**Table 8:** Impact of good quality and formal sources of seed on rice yield across farm categories by propensity score matching methods.

Matching method and outcome via ATT	Farm category			
	Small	Medium	Large	All
**Impact of good quality seed on rice yield (ton/ha)**				
Nearest neighbour matching	0.16* (0.09)	0.07* (0.09)	0.06* (0.07)	0.28*** (0.08)
Kernel matching	0.17** (0.06)	0.04* (0.07)	0.11** (0.08)	0.14*** (0.04)
Radius matching at 5%	0.11** (0.05)	0.05* (0.05)	0.14** (0.09)	0.09*** (0.04)
Radius matching at 10%	0.09** (0.04)	0.06* (0.05)	0.18*** (0.07)	0.07*** (0.03)
Stratification matching	0.10 (0.11)	0.03 (0.11)	0.21 (0.16)	0.10 (0.09)
**Impact of formal sources of seed on rice yield (ton/ha)**				
Nearest neighbour matching	0.07** (0.06)	0.09* (0.06)	0.14* (0.10)	0.15*** (0.06)
Kernel matching	0.04* (0.03)	0.05* (0.09)	0.11* (0.07)	0.05** (0.05)
Radius matching at 5%	0.02* (0.03)	0.04* (0.08)	0.15** (0.13)	0.03* (0.03)
Radius matching at 10%	0.11** (0.08)	0.05** (0.05)	0.19* (0.09)	0.03* (0.02)
Stratification matching	0.14*** (0.03)	0.05** (0.02)	0.22** (0.09)	0.06 (0.12)

ATT means average treatment of treated. The figure in the parentheses indicates robust standard errors. Significance level

*** p<0.01

** p<0.05

* p<0.10

About formal seed sources, farmers with greater access to such sources achieved average yield increases ranging from 0.03 t/ha (p<0.10) to 0.15 t/ha (p<0.01) compared to yields of farmers relying on informal seed sources in the study areas. Across different farm categories and matching algorithms, small farmers with greater access to formal seed sources experienced average yield increases ranging from 0.02 t/ha (p<0.10) to 0.14 t/ha (p<0.01), medium farmers observed increases ranging from 0.04 t/ha (p<0.10) to 0.09 t/ha (p<0.10), and large farmers with greater access to formal seed sources witnessed increases ranging from 0.11 t/ha (p<0.10) to 0.22 t/ha (p<0.05). The results consistently indicated that the positive effects (yield improvement) of good quality seed usage and access to formal seed sources were more pronounced among small-scale farmers in the study areas.

## 5. Discussion

**Fig 3:**
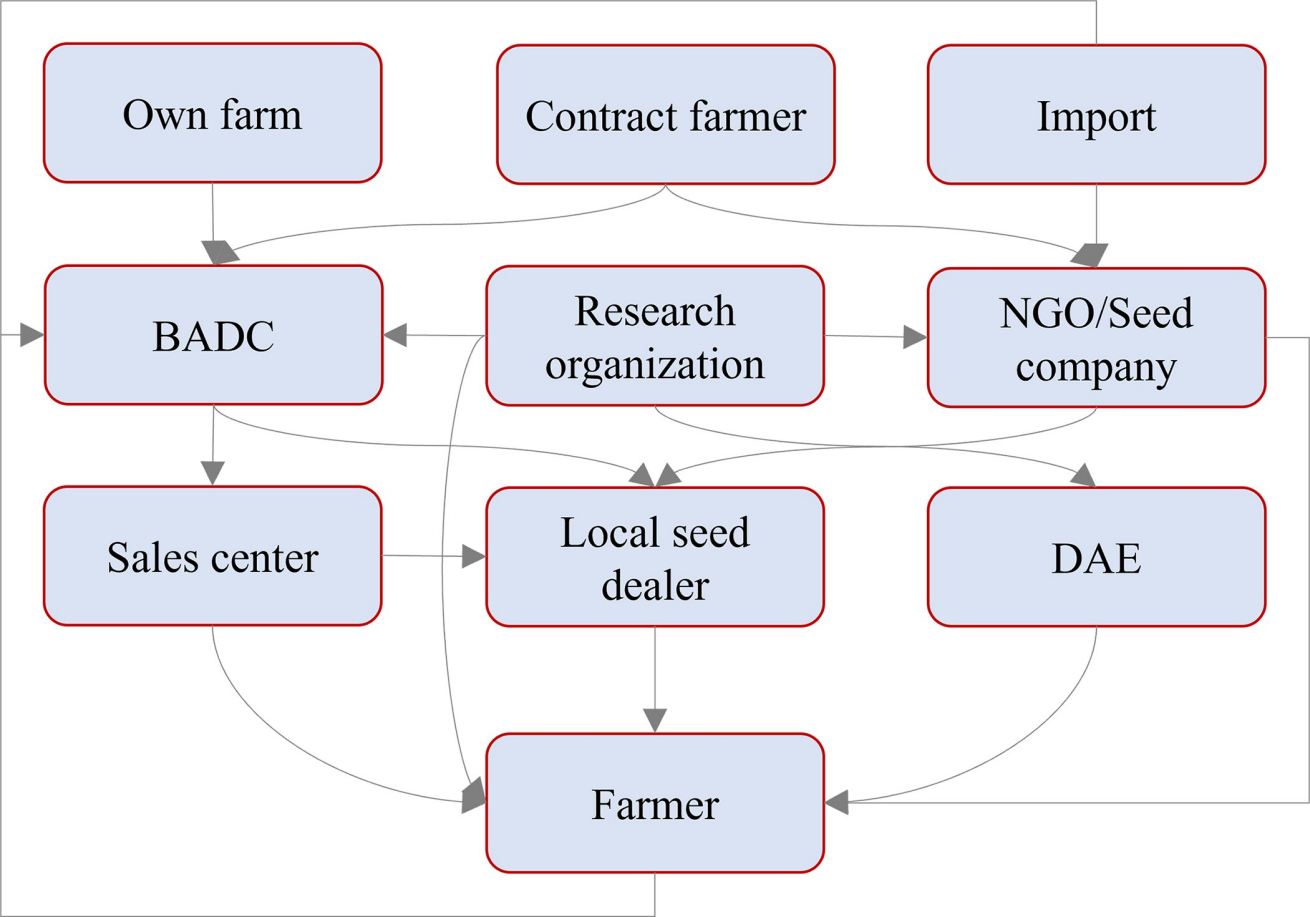
Formal rice seed system network in Bangladesh. Source: Prepared by authors based on data from field observation.

Seeds are the foundation for crop production. Quality seeds are essential for producing healthy, nutritional, and high-yielding crops. In Bangladesh, about 91.70% of farmers are smallholders [72], and they struggle with economic poverty and encounter numerous barriers to accessing a resilient and inclusive seed system. From our observation, the formal rice seed system in the northern region of Bangladesh (Fig 3) consists of three main actors: the public sector, the private sector, and non-governmental organizations (NGOs) [9, 14]. The majority of rice seed actors engage in seed production through their own farms or by partnering with contract growers. Additionally, they rely on importing seeds from other countries to meet farmers’ requirements. One farmer in the study areas reported that the Bangladesh Agricultural Development Corporation (BADC) sometimes collects seeds directly from farmers; in most cases, the seeds are of poor quality. The frequent occurrence of farmers receiving poor quality adulterated rice seed from BADC raises concerns about the efficiency and trust of the public seed system. However, the channels utilized by farmers differ based on the resources accessible in their local area, the assortment of crops and varieties they cultivate, and various economic, social, cultural, and political factors that impact decision-making at both the individual and household levels [13, 61]. Only the public sector seed distributor, BADC, disseminates seeds through its sales centers and local seed vendors [9, 73]. On the other hand, research organizations develop and disseminate new seed varieties through the Department of Agricultural Extension (DAE), field experiments, BADC, NGOs, and seed companies. Private sectors distribute seeds through their registered dealers, while NGOs utilize their extending networks to provide seeds either by farmers’ groups or through sales personnel. So altogether, the formal seed system supplies about 32% of quality seed to farmers, with specific percentages varying across different crops, such as 65% for rice, 45% for wheat, 74% for maize, and 16% for potato [6]. Farmers in the informal seed network obtain rice seeds through various means, including their own production, seed exchanges with other farmers, accessing local markets, and also from the border market [2, 13, 74, 75]. So, seed traders exert a substantial influence on farmers, contributing to the increased use of good quality seeds through the dissemination of knowledge, promotion of new varieties, and encouragement of a market-driven seed system [53].

We found that about 34% of farmers used public seed sources, 33% used private seed sources, and the remaining farmers used their own seeds (Refer to Table 3). Generally, the formal seed systems actors tend to prioritize the requirements of commercial or large farmers; consequently, smallholder farmers often depend on farm-saved and local market seeds of poor quality. A study conducted in six African countries found that 90.2% of seed obtained by producers comes from informal systems [24]. Local seed systems play a crucial role in enhancing seed security for resource poor farmers and promoting crop diversification [76]. About 50% of Ugandan farmers used their own farm-saved seed [22], while 40% of Ethiopian farmers used wheat seed from the formal sector [77]. But our study reveals divergent findings, as small farmers have better access to formal seed systems and use good quality seed. There are several reasons for this. Firstly, most small farms in Bangladesh operate on a subsistence basis, selling their produce immediately after harvest to sustain their livelihoods. This leaves them unable to retain seed for the following season. Secondly, small farmers have well-established connections with seed dealers, enabling them to easily meet their variety-specific demands. Lastly, due to limited knowledge and experience regarding the production and storage capabilities of quality seed (Refer to Table 2), small farmers tend to exhibit risk-averse behavior. In terms of yield advantage, our result of the PSM model revealed that good quality seed and seed from formal sources increased rice yields significantly (Refer to Table 8). Likewise, studies conducted in Nepal and Kenya demonstrated the positive impact of certified seeds on revenues, profits, and yields [56, 78].

From a preference perspective, our study observed a 48% gap in accessing good quality seed (Refer to Table 3). Remarkably, small farmers displayed a relatively greater preference for quality seed compared to other farm categories, which was consistent with findings from Nepalese farmers [79]. Furthermore, in India and Bangladesh, women’s self-help groups have collaboratively initiated and propagated quality seed practices for rice and wheat varieties [16, 55]. We also found that farmers’ preferences regarding packet size and type influenced the adoption and diffusion of seeds. All category farmers preferred *polycoated* jute sacks over plastic packets for long-term storage, trust in ensuring higher germination rates, and avoiding mishandling. In Ethiopian studies, it was observed that hermetic bags effectively preserve the quality of rice seeds [36], whereas the use of polypropylene bags was associated with a decline in seed quality [80]. More specifically, most Ethiopian farmers (78%) used the *Gotera* storage structure, while only 13.2% opted for jute bags, and 8.1% opted for polypropylene bags to store their seeds [77]. According to our study findings, farmers showed a preference for 5 kg rice seed packets (Refer to Table 3). This preference can be attributed to the predominance of small farm holdings and the cultural practice of cultivating diverse crops and varieties within a single season. As a result, allocating a larger portion of land for a single crop rice seed may not align with their agricultural practices. In comparison with other countries, legume seeds in Zambia were packaged in 50 kg bags, while in Guatemala, potato seeds were available in 22 kg/50 pounds wooden boxes or bags of 45 kg/100 pounds, and bean seeds were offered in packages ranging from 3, 5, 25, and 100 pounds [49]. However, the study did not ascertain farmers’ preferences for packet sizes, as this varied according to crop type and geographical regions.

Seed sector has high degree of dynamism and uncertainty [81]. Farmers exhibit a strong reliance on traditional varieties and are more hesitant to engage in experimentation with newer alternatives [82]. The behaviour of rice farmers in the northern region of Bangladesh in selecting seeds changed based on their anticipation of achieving higher yields (Refer to Table 4) and obtaining seeds at a lower price (Refer to Table 6). Similar to this study result, Tanzanian, Ghanaian, Nepalese, and Chinese producers were considerably more likely to pay a premium price for high-quality certified seeds [79, 83, 84]. Kenyan farmers were paid a 15% higher price for bags of seeds purchased directly from the seed company than local dealers [20].

We explored the determinants of rice farmers’ preferences for quality seed, packet types, sizes, and seed sources in northern Bangladesh through ordered logit and multinomial logit regressions (Refer to Table 7). Heterogeneous factors were identified, encompassing demographic, farm, infrastructure, and market characteristics. Moreover, supply system challenges such as the limited availability of quality seeds, restricted access to seed markets, high seed prices, insufficient information on seed attributes and sources, unavailability of desired seeds, hassles to get seed, impure seeds, and distance to seed sources significantly altered rice farmers’ preferences for seed quality, type, size, and source. Similar findings have been found in past literatures [2, 22, 24, 25, 43, 56, 78, 84]. Beyond the above-mentioned factors, Hossain and Ahmed [14] identified key challenges facing the supply of quality seeds in Bangladesh, such as the lack of accurate statistics on cultivable land for different crops, coupled with inadequate seed production and marketing plans; the absence of a mechanism to assess and forecast seed demand; high and unjustified seed prices that limit the access of resource-poor farmers to quality seeds; instability in the prices of farmers’ produce; and limited capacity of BADC and Seed Certification Agency (SCA), which are responsible for seed supply and quality control regulations in the country.

In our study, it was evident that farmers expressed dissatisfaction with the prevailing sales prices of rice seeds in the public and private sources (Refer to Tables 2 and 6). In Bangladesh, the production cost of one kilogram of rice seed ranges from 32–35 BDT, considering factors such as processing and transportation costs [41]. However, the sales price of rice seeds ranges from 41–49 BDT/kg (Refer to Table 5). Consequently, market actors are able to obtain a net margin ranging from 9–14 BDT/kg. To bridge this dissatisfaction gap, two potential approaches can be considered. Firstly, the government could contemplate subsidizing the cost of rice seeds, thereby making them more affordable for farmers. Secondly, efforts could be made to reduce rice seed production costs through the adoption of mechanization and the provision of support inputs.

### 6. Policy considerations, study limitations, and future prospects

The study highlights the importance of reliable seed sources in increasing rice yields and recommends continued government support to strengthen formal seed supply chains. Additionally, a reliable and competitive seed market facilitates farmer to access a wide range of desired rice seed varieties and qualities, thereby promoting the adoption of good quality seeds. Policymakers and market actors are urged to focus on creating an environment for robust seed markets, including measures to promote competition, quality assurance, and market transparency. Meeting farmers’ needs like credit access and tailoring interventions to address their challenges fosters inclusive agricultural development.

The study has important implications for achieving the Sustainable Development Goals (SDGs), specifically target 2.3 of doubling agricultural productivity by 2030. By promoting the adoption of good quality seed and formal seed sources among farmers, policymakers and stakeholders can support the goal of achieving food security and reducing hunger. Increased agricultural productivity can enhance the availability and affordability of nutritious food, thus improving the overall nutrition status of communities. The study’s implications also align with SDG 12, emphasizing sustainable agricultural practices and the efficient use of resources. The use of good quality seeds can contribute to sustainable agriculture by optimizing resource utilization, reducing wastage, and minimizing the environmental impact of farming activities. Additionally, SDG 17 underscores the significance of multi-stakeholder collaborations and knowledge exchange for SDGs attainment. By fostering partnerships between governments, seed companies, research institutions, and farmers’ organizations, it becomes possible to promote the adoption of good quality seed, strengthen formal seed supply chains, and develop inclusive and resilient seed systems.

To effectively connect these findings with the SDG targets, it is crucial to integrate them into policy frameworks and action plans. Governments should prioritize investments in agricultural research and extension services to enhance farmers’ awareness and understanding of seed quality, formal seed sources, and market preferences. This can be achieved through the development of tailored training programs, the dissemination of information materials, and the establishment of farmer field schools to promote best practices in seed selection and utilization. Additionally, efforts should be made to improve seed infrastructure, including the establishment of seed testing laboratories, quality control mechanisms, and seed certification systems at the root level. This can ensure the availability of good quality seed and facilitate farmers’ access, especially small-scale farmers, to reliable formal seed sources.

No study is exempt from limitations, and our research is no exception. This study primarily analyzes factors influencing rice farmers’ perceptions of good quality seeds usage, access to formal seed sources, and packet characteristics, considering demographic, farm, and market factors. The study overlooked psychological influences on farmers’ decision-making. Future studies could explore the role of factors like risk perception, attitudes, beliefs, and decision-making biases in shaping farmers’ choices concerning seed usage, sources, and market preferences. Another limitation of this study is the lack of empirical evidence demonstrating the direct impact of seed package size on yield, suggesting a need for future research to experimentally investigate this relationship to provide more comprehensive insights. This study relied on cross-sectional data. Future studies should utilize panel data analysis to comprehensively capture long-term effects and dynamics. This analytical approach would enable a more thorough exploration of changes over time and facilitate a deeper understanding of the causal relationship between seed quality, formal sources, market branding, and rice yield. Moreover, conducting comparative studies across different regions or farming systems, along with adoption assessments and socioeconomic impact evaluations, can contribute to a better understanding of effective strategies for promoting the use of good quality seeds and formal seed sources. By addressing these limitations and pursuing further research avenues, valuable insights can be gained to inform agricultural practices and guide policy development.

## 7. Conclusions

This study aimed to address the existing research gap concerning the significance of farmers’ preferences in establishing an efficient rice seed system. We found a notable gap in the utilization of quality seeds and limited access to formal seed sources across farm categories. Addressing the gap in good quality seed usage and establishing a well-connected network for accessing formal seed sources is crucial for fostering inclusivity among farms and enhancing rice productivity. Furthermore, farmers in the study areas displayed a preference for *polycoated* jute sacks and 5 kg size packets of rice seed. The significance of farmers’ preference for 5 kg size packets extends to the realm of the SDGs, as it directly contributes to enhancing the livelihoods of smallholder farmers and informs the development of effective market strategies. Additionally, the preference for *polycoated* jute sacks among farmers revealed a growing consciousness regarding the utilization of eco-friendly products. These findings carry substantial environmental advantages, including the reduction of plastic waste, the lowering of carbon emissions, the promotion of sustainable practices, as well as the support for the local jute industry and the livelihoods of individuals engaged in jute cultivation and processing. The study also identified heterogeneous factors influencing rice farmers’ seed preferences, providing valuable insights into the complex seed system in highly crop-intensive areas of Bangladesh. This knowledge can inform targeted interventions and policy measures aimed at promoting improved seed utilization and strengthening rice seed systems. In conclusion, our study highlights the importance of considering farmers’ preferences in the design and implementation of seed-related initiatives that foster sustainable agricultural development.

## Supporting information

S1 Data (XLS)

S1 Table The parameter estimates of the ordered logit regression for quality seed and packet size, and the multinomial logit regression for packet types and seed sources. (DOCX)
