# Supplementary material for: Rice farmers’ preferences for seed quality, packaging, and source: A study from northern Bangladesh
Source: PLoS One. 2024 Jun 21;19(6):e0306059. doi: 10.1371/journal.pone.0306059 (PMC11192390; doi:10.1371/journal.pone.0306059)
Supplement: S1 Table — (DOCX) [file pone.0306059.s002.docx]

**Table S1**. **The parameter estimates of the ordered logit regression for quality seed and packet size, and the multinomial logit regression for packet types and seed sources.**

| Parameters | Ordered logit regression | | Multinomial logit regression | | | |
| --- | --- | --- | --- | --- | --- | --- |
|  | Quality seed | Packet size | Packet types (ref. plastic packet) | | Seed sources (ref. farm-saved) | |
|  |  |  | Polycoated jute sack | Plastic sack | Private | Public |
| Farmers’ age (years) | -0.023 (0.04) | 0.047* (0.03) | -0.098 (0.07) | -0.030 (0.10) | 0.064 (0.04) | -0.014 (0.09) |
| Farmers’ age squared (years) | 0.0004 (0.001) | -0.0004 (0.001) | 0.012* (0.007) | 0.0003 (0.001) | -0.001 (0.0004) | 0.0002 (0.001) |
| Farmers’ education (years) | 0.005 (0.01) | 0.004 (0.01) | 0.012* (0.006) | -0.072*** (0.02) | 0.031* (0.02) | 0.036* (0.02) |
| Gender (1=male) | -0.272* (0.16) | 0.336** (0.15) | -0.499*** (0.14) | 0.518*** (0.17) | 0.102 (0.22) | -0.395 (0.38) |
| Small farms (ref. large) | 1.332*** (0.40) | -0.415 (0.37) | 1.032* (0.59) | -0.630* (0.33) | -1.423*** (0.47) | 1.939** (0.89) |
| Medium farms (ref. large) | 0.724** (0.31) | -0.446 (0.30) | 0.407 (0.77) | -0.899*** (0.25) | -0.966*** (0.37) | 1.229* (0.73) |
| Operated land in decimal (logarithm) | -0.228* (0.12) | 0.013 (0.11) | 0.164 (0.23) | 0.151 (0.30) | -0.148 (0.16) | 0.106 (0.28) |
| Distance from home to seed dealers (km) | -0.022** (0.01) | 0.033*** (0.01) | -0.050*** (0.02) | -0.057 (0.04) | -0.019** (0.01) | -0.015* (0.01) |
| Distance from home to Upazila office (km) | -0.007 (0.01) | -0.020** (0.01) | -0.050*** (0.01) | 0.030* (0.02) | -0.016 (0.01) | -0.030 (0.02) |
| Access to public seed sources (ref. farm-saved) | 1.445*** (0.32) | 0.838** (0.34) | 0.875** (0.38) | -1.651** (0.66) | - | - |
| Access to private seed sources (ref. farm-saved) | 0.070 (0.16) | 0.509*** (0.15) | -0.147 (0.28) | 0.194* (0.11) | - | - |
| Advice from public dealers (ref. other farmers’) | 0.878*** (0.19) | 0.903*** (0.17) | 0.281 (0.36) | -0.142 (0.55) | -0.221 (0.23) | 0.636*** (0.16) |
| Advice from private dealers (ref. other farmers’) | 2.026*** (0.23) | 0.800*** (0.20) | 0.012 (0.39) | 0.818* (0.48) | 0.080** (0.04) | 2.472*** (0.54) |
| Advice from extension office (ref. other farmers’) | 1.042*** (0.22) | 0.715*** (0.18) | 0.234 (0.42) | 1.278*** (0.45) | 0.207 (0.29) | 0.379* (0.21) |
| Adulterated seed (1=yes) | -2.469*** (0.23) | -0.240** (0.12) | -0.444** (0.21) | -0.730* (0.39) | -0.195 (0.28) | -0.318** (0.14) |
| Seed is costly (1=yes) | -0.764** (0.32) | 0.006 (0.34) | -1.258** (0.60) | -1.521** (0.67) | -1.351*** (0.13) | -1.449*** (0.17) |
| Pay more than fixed price (1=yes) | -0.525*** (0.19) | 0.091 (0.19) | -0.338 (0.40) | 0.007 (0.45) | -0.031 (0.23) | -0.453 (0.81) |
| Unavailability of desired seed (1=yes) | -0.735** (0.30) | 0.735*** (0.25) | 0.512 (0.58) | 1.042 (0.64) | -0.024* (0.01) | -0.207** (0.09) |
| Available but not in time (1=yes) | -0.063* (0.04) | 0.130 (0.20) | -0.307 (0.44) | 0.269 (0.52) | -0.364 (0.30) | -0.160 (0.93) |
| Hassles to get seed (1=yes) | -0.466** (0.21) | 0.460* (0.27) | -0.926* (0.49) | -0.566 (0.56) | 0.442* (0.25) | -2.156** (1.04) |
| Lack of knowledge about quality seed (1=yes) | -0.742*** (0.27) | -0.139 (0.23) | -1.146* (0.60) | -0.852 (0.71) | 0.220 (0.32) | -1.213 (1.03) |
| Dissatisfaction with the price paid (1=yes) | -1.673*** (0.31) | -0.081 (0.24) | 0.493 (0.63) | 0.341 (0.74) | -0.164 (0.33) | -2.356** (1.02) |
| Rice seed sales price (BDT/kg) | -0.063 (0.05) | -0.029 (0.05) | -0.378*** (0.08) | -0.239** (0.10) | -0.074 (0.07) | -0.200* (0.11) |
| Price difference between expected and sales prices of rice (BDT/kg) | -0.035 (0.05) | -0.048 (0.05) | -0.325*** (0.08) | -0.244** (0.10) | -0.079 (0.06) | -0.276*** (0.10) |
| Quantity of rice seed used in hectares (logarithm) | -0.056*** (0.02) | 0.021 (0.02) | 0.015 (0.03) | -0.007 (0.04) | -0.096*** (0.02) | -0.090** (0.05) |
| Squared quantity of rice seed used in hectares (logarithm) | -0.001* (0.002) | 0.0001 (0.0003) | 0.0001 (0.0004) | -0.0001 (0.001) | -0.001*** (0.0003) | -0.001 (0.001) |
| Rice yield (ton/ha) | 0.242* (0.13) | - | 0.375** (0.18) | -0.599* (0.34) | 0.182 (0.19) | 0.291 (0.31) |
| Polycoated jute sack (ref. plastic packet) | - | 1.753*** (0.24) | - | - | -0.028 (0.28) | 1.233* (0.66) |
| Plastic sack (ref. plastic packet) | - | 0.819*** (0.28) | - | - | 0.028 (0.34) | -1.317* (0.77) |
| 1 kg packet seed (ref. 10 kg) | - | - | -3.020*** (0.48) | -1.504** (0.64) | -1.112*** (0.43) | -1.154** (0.50) |
| 2 kg packet seed (ref. 10 kg) | - | - | -2.021*** (0.41) | 0.120*** (0.03) | -0.753*** (0.27) | -1.672** (0.83) |
| 5 kg packet seed (ref. 10 kg) | - | - | -0.944** (0.39) | 0.158*** (0.05) | -0.654*** (0.21) | -0.216 (0.47) |
| Good quality seed (ref. poor) | - | - | - | - | 0.046* (0.03) | 2.334*** (0.62) |
| Average quality seed (ref. poor) | - | - | - | - | 0.340** (0.16) | 0.761 (0.59) |
| Intercept (cut1) | -1.576* (0.83) | -1.646** (0.76) | 2.008*** (0.77) | 1.870*** (0.62) | -3.415*** (0.14) | 2.067 (3.80) |
| Intercept (cut2) | 0.620** (0.26) | 0.323* (0.18) | - | - | - | - |
| Intercept (cut3) | - | 0.825* (0.43) | - | - | - | - |
| Pseudo r-squared | 0.25 | 0.22 | 0.30 | | 0.24 | |
| Wald chi-square | 385.62*** | 309.56*** | 318.56*** | | 598.90*** | |
| Akaike criteria (AIC) | 1866.42 | 2479.31 | 1200.52 | | 1349.83 | |
| Bayesian criteria (BIC) | 2013.94 | 2636.99 | 1515.90 | | 1675.38 | |
| Number of observations | 1196 | 1196 | 1196 | | 1196 | |

The figure in the parentheses indicates robust standard errors. Significance level: **** p<0.01, ** p<0.05, * p<0.10*
